# Ultrafast thermo-optical control of spins in a 2D van der Waals semiconductor

**DOI:** 10.1038/s41467-025-58065-1

**Published:** 2025-03-21

**Authors:** Maciej Da̧browski, Sumit Haldar, Safe Khan, Paul S. Keatley, Dimitros Sagkovits, Zekun Xue, Charlie Freeman, Ivan Verzhbitskiy, Theodor Griepe, Unai Atxitia, Goki Eda, Hidekazu Kurebayashi, Elton J. G. Santos, Robert J. Hicken

**Affiliations:** 1 Department of Physics and Astronomy, University of Exeter, Exeter, EX4 4QL United Kingdom; 2 Institute for Condensed Matter Physics and Complex Systems, School of Physics and Astronomy, The University of Edinburgh, Edinburgh, EH9 3FD United Kingdom; 3 London Centre for Nanotechnology, University College London, 17-19 Gordon Street, London, WCH1 0AH UK; 4 Department of Physics, National University of Singapore, 2 Science Drive 3, Singapore, 117542 Singapore; 5 Instituto de Ciencia de Materiales de Madrid, CSIC, Cantoblanco, 28049 Madrid, Spain; 6 Centre for Advanced 2D Materials, National University of Singapore, 6 Science Drive 2, Singapore, 117546 Singapore; 7 Department of Chemistry, National University of Singapore, 3 Science Drive 3, Singapore, 117543 Singapore; 8 Department of Electronic & Electrical Engineering, UCL, London, WC1E 7JE United Kingdom; 9 WPI Advanced Institute for Materials Research, Tohoku University, 2-1-1, Katahira, Sendai, 980-8577 Japan; 10 Higgs Centre for Theoretical Physics, University of Edinburgh, Edinburgh, UK; 11 Donostia International Physics Center (DIPC), 20018 Donostia-San Sebastián, Basque Country, Spain

**Keywords:** Two-dimensional materials, Magnetic properties and materials, Spintronics

## Abstract

Laser pulses provide one of the fastest means of manipulating electron spins in magnetic compounds and pave the way to ultrafast operation within magnetic recording, quantum computation and spintronics. However, effective management of the heat deposited during optical excitation is an open challenge. Layered two-dimensional (2D) van der Waals (vdW) materials possess unique thermal properties due to the highly anisotropic nature of their chemical bonding. Here we show how to control the rate of heat flow, and hence the magnetization dynamics, induced by an ultrafast laser pulse within the 2D ferromagnet Cr_2_Ge_2_Te_6_. Using time-resolved beam-scanning magneto-optical Kerr effect microscopy and microscopic spin modelling calculations, we show that by reducing the thickness of the magnetic layers, an enhancement of the heat dissipation rate into the adjacent substrate leads to a substantial reduction in the timescale for magnetization recovery from several nanoseconds down to a few hundred picoseconds. Finally, we demonstrate how the low thermal conductivity across vdW layers may be used to obtain magnetic domain memory behaviour, even after exposure to intense laser pulses. Our findings reveal the distinctive role of vdW magnets in the ultrafast control of heat conduction, spin dynamics and non-volatile memory.

## Abstract (web summary)

The layered structure of van der Waals materials leads to highly anisotropic thermal conductivity, due to the van der Waals gap between the layers. Here, Da̧browski et al show how this anisotropic heat transport can be harnessed for ultrafast, optically-induced control of magnetism in Cr_2_Ge_2_Te_6_.

## Introduction

Controlling the thermal conductivity of a material is one of the biggest challenges in modern electronics. As the characteristic dimensions of devices shrink to the nanoscale, increased heat dissipation is of critical importance, limiting device effectiveness and overall feasibility^1^. The strong in-plane covalent bonds and weak vdW interactions between the layers give most 2D materials unique anisotropic thermal conductivity properties, that are inaccessible in other materials^2,3^. While there are numerous reports of heat transfer in non-magnetic vdW structures^3–5^, little is known about how the heat is absorbed and stored by spins in the recently discovered vdW magnets^6,7^. Spins provide additional degrees of freedom and new functionalities^8^ that can be controlled on ultrafast timescales via laser pulses^9^. Recent studies of 2D magnets revealed that laser pulses can control gate-tunable spin waves^10^, induce coherent spin-phonon oscillations^11^, generate nontrivial topological spin textures^12–14^, launch exciton-coupled coherent magnons^15^, and switch the magnetization direction^16,17^. All of these processes are inextricably associated with additional, optically-induced heat being deposited into the sample.

Since the first observation of ultrafast demagnetization^18^, significant progress has been made in understanding its underlying microscopic origin^19,20^. In contrast, considerably less attention has been paid to remagnetization processes, i.e., the recovery of magnetization after exposure to an ultrafast laser pulse^21,22^. Heat conduction is the primary process governing remagnetization and remains a key engineering challenge in real-world applications such as heat-assisted magnetic recording (HAMR)^23^. Thanks to the directional dependence of heat transport, and the ability to combine various materials without lattice-matching constraints, 2D vdW materials are expected to surpass conventional materials in thermal management of future electronic and spintronic devices^24^. Despite enormous interest in vdW magnets^8^, the dynamics of remagnetization and heat dissipation following excitation by a laser pulse are yet to be unveiled.

**Fig. 1:**
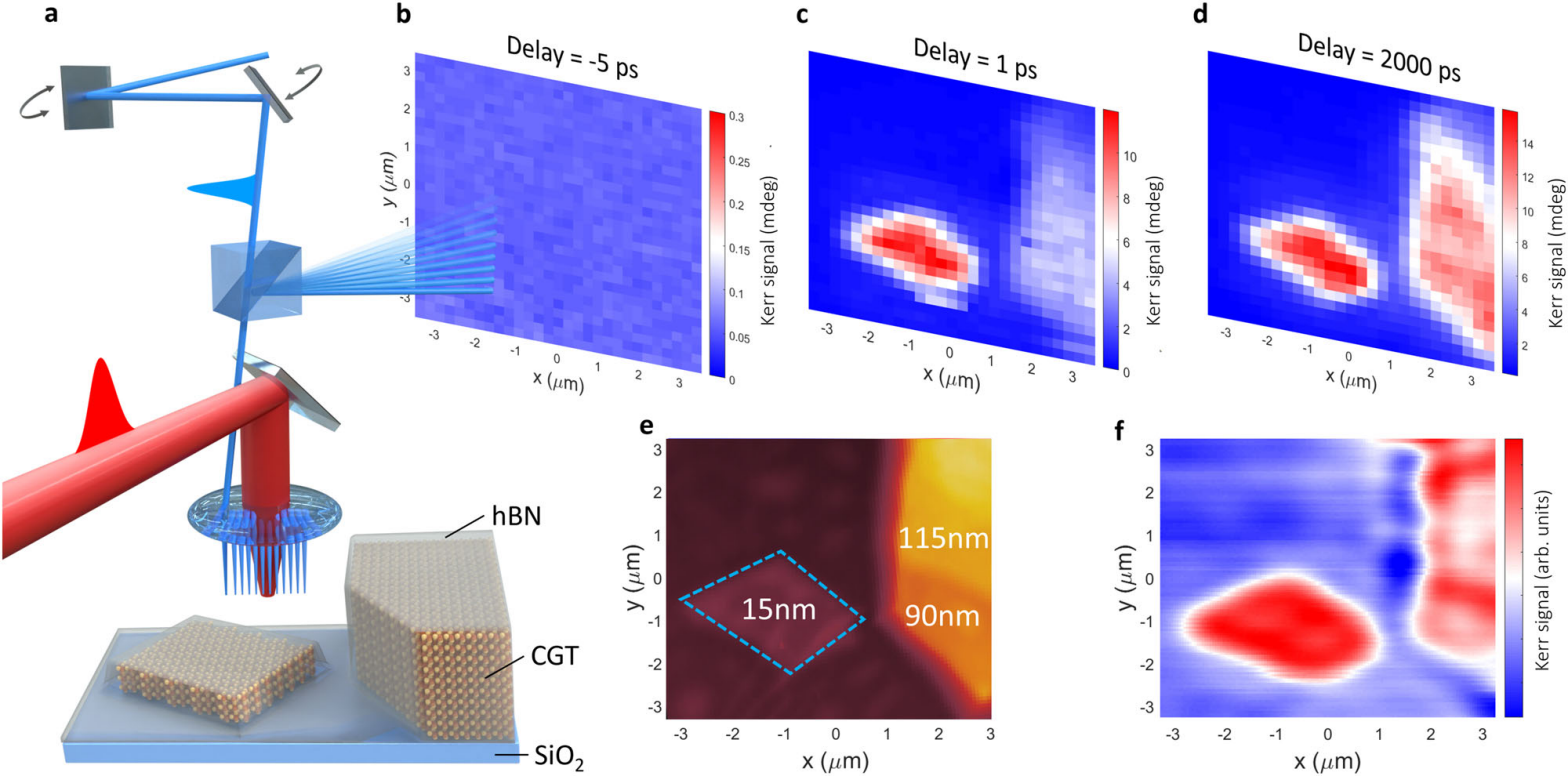
Time-resolved imaging of magnetization dynamics by means of beam-scanning microscopy. **a** Schematic representation of the beam-scanning MOKE microscopy technique. Two galvo mirrors are used to scan the position of the 400 nm probe beam (blue color) on the sample. Delaying the probe with respect to the 800 nm pump (red beam) enables simultaneous time-resolved imaging of 2D flakes with different thicknesses. **b**–**d** Time-resolved images acquired at time delays of -5, 1 and 2000 ps, respectively, for CGT flakes with thickness of 15 nm, 90 nm and 115 nm as shown in the AFM image in (**e**). **f** Static MOKE image acquired by scanning the probe beam over the same area as **b**–**e**. The measurements were performed at 6 K with magnetic field *μ*_0_*H* = 0.5 T applied along the sample normal.

Here, we directly probe the laser-induced magnetization dynamics in the 2D vdW magnet Cr_2_Ge_2_Te_6_ (CGT) using time-resolved beam-scanning magneto-optical Kerr effect (MOKE) microscopy (see Fig. 1a and Methods). By varying the CGT thickness, we demonstrate how to tune the timescale of the remagnetization process via modification of the heat dissipation rate into the adjacent SiO_2_/Si substrate which acts as a heat sink. We reveal that the low thermal conductivity across the layers prevents the formation of a random domain structure at remanence, even under pumping conditions that lead to a full loss of magnetic order in the layers directly probed by the laser excitation. Instead, the exact same domain pattern is restored in the top layers after demagnetization, owing to the stray field from the layers beneath. This allows thick CGT to act as a robust magnetic domain memory (MDM)^25^ under the influence of intense laser pulses, a behavior that has so far been unachievable in other materials.

## Results

To probe the ultrafast magnetization dynamics with sub-micron spatial resolution a two-color pump-probe beam-scanning MOKE microscopy technique was developed. Here the probe beam (*λ* = 400 nm) is scanned relative to the pump beam (*λ* = 800 nm) using a galvanometer scanner and the time delay between pump and probe pulses is carefully controlled by means of a standard optical delay line (Fig. 1a and Supplementary Fig. 1). In this geometry, the dynamic MOKE signal measured with a balanced photodiode detector is proportional to the transient change of the magnetization component perpendicular to the sample plane (*Δm*_z_). Time-resolved images at a fixed time delay of -5 ps (Fig. 1b), 1 ps (Fig. 1c) and 2000 ps (Fig. 1d) show the transient Kerr signal proportional to the reduction of the magnetization (demagnetization) with respect to the ground state, which for the 0.5 T field applied along the sample normal is the saturated mono-domain state (see static MOKE image in Fig. 1f for the ground state). Probing different thicknesses of CGT simultaneously allows differences in the magnetization dynamics between thinner 15 nm and thicker 90 nm and 115 nm flakes to be identified immediately (see the Atomic Force Microscopy (AFM) image in Fig. 1e and Supplementary Section S2 for positions of the flakes). In particular, after 1 ps the Kerr signal is much larger for the thinner 15 nm flake as compared to the thicker flakes, while it is similar for all thicknesses after 2000 ps. Note that here the pump beam is slightly defocused, to ensure homogeneous excitation in the pump-probe measurements, in contrast to previously used beam-scanning setups^15,26^. For measurements shown in Fig. 1b–d the pump beam is focused to a 50 *μ*m diameter spot (intensity at 1/*e*^2^) using a  × 20 objective lens, as measured by a beam profiler at the focal plane of the probe beam. A detailed discussion on the effect of the pump beam profile can be found in the Supplementary Section S1.

**Fig. 2:**
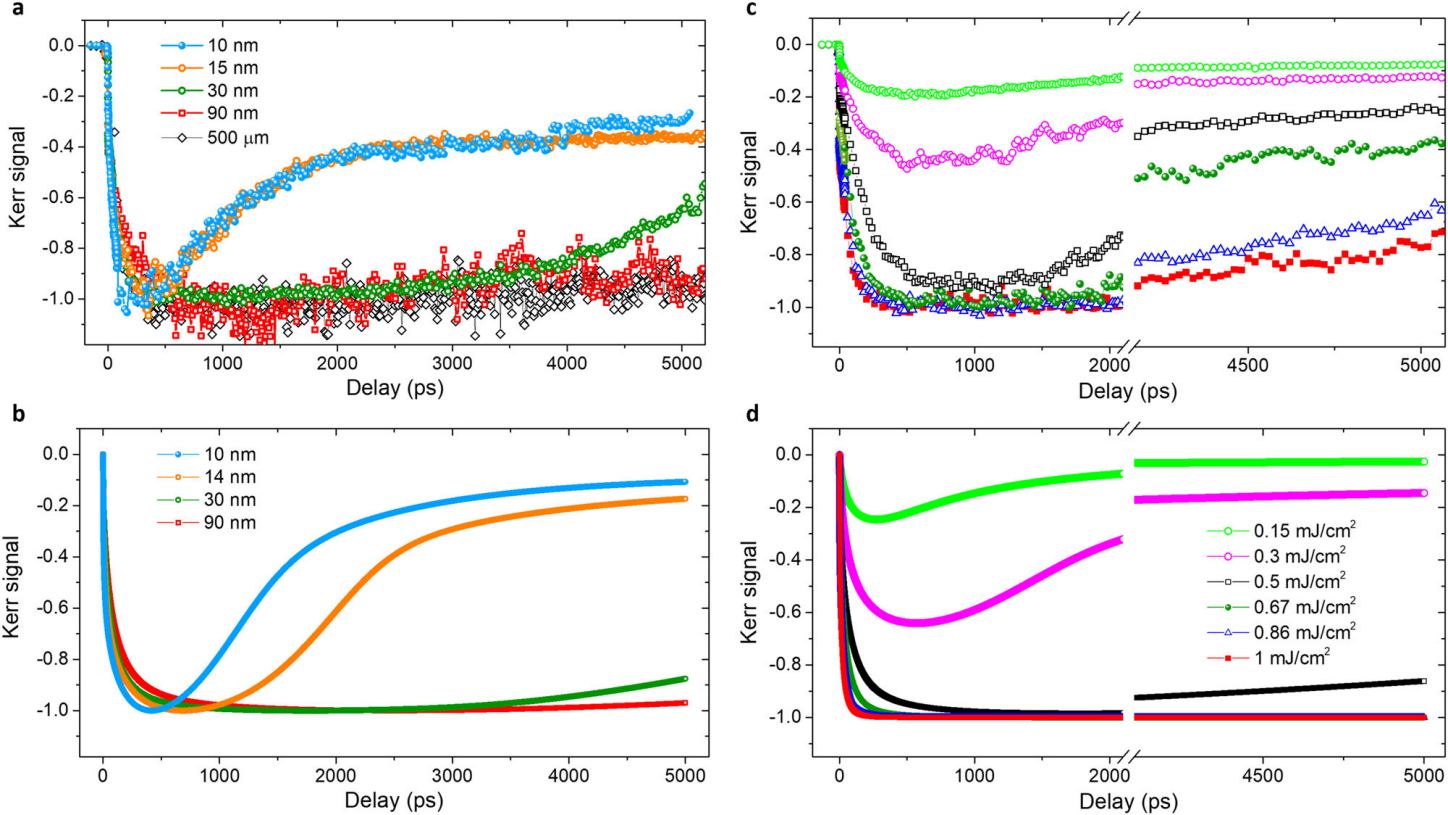
Thickness and fluence dependent spin dynamics. **a** Normalized Kerr signal for different thicknesses of CGT flakes measured by TR-MOKE at *F* = 0.86 mJ/cm^2^. **b** Calculated Kerr signal for different CGT thicknesses. **c, d** Fluence dependence for 30 nm CGT flake from experiment (**c**) and theory (**d**). The measurements were performed at 6 K and with *μ*_0_*H* = 0.5 T.

Next, systematic time-resolved measurements were made on separate CGT flakes with different thicknesses: 10 nm, 15 nm, 30 nm, 90 nm and 500 *μ*m. All flakes, except for the 500 μm bulk crystal, were exfoliated onto the Si/SiO_2_ substrate and encapsulated with hBN (see Methods). In order to compare the dynamic Kerr signal from different flakes, the Kerr signal was normalized to the full demagnetization, which corresponds to a complete loss of magnetic order (see Supplementary Section S4). The time-resolved MOKE (TR-MOKE) traces (Fig. 2a) were acquired at fixed pump and probe position, in the centre of each flake, in an applied field of 0.5 T to ensure the saturation of all magnetic moments. Starting with the thickest 500*μ*m sample, full demagnetization is observed and persists until 6000 ps, the maximum delay time available within the experiment, which to the best of our knowledge is the longest remagnetization process observed so far. This is in agreement with a previous single color 800 nm pump-probe study where a bulk-like flake showed no signs of remagnetization up to 3500 ps^27^. The 90 nm thick flake follows exactly the same trend as the 500 *μ*m bulk crystal, demonstrating that neither the top hBN layer nor the Si/SiO_2_ substrate significantly affects the magnetization dynamics in this case. With a further decrease of the flake thickness down to 30 nm, remagnetization beyond about 3500 ps is observed. Finally, for the thinnest flakes, 15 nm and 10 nm, remagnetization begins immediately after full demagnetization is achieved, i.e., after  ~ 400 ps. The TR-MOKE signal is very similar for 15 nm and 10 nm suggesting that further reduction in thickness below 15 nm does not substantially alter the magnetization dynamics.

To gain more insight into the experimental observations, microscopic spin modeling calculations have been performed. A semi-classical three-temperature model (3TM)^28^ was used to simulate the thermal transport during the ultrafast laser heating and the magnetization dynamics for different delay times and thicknesses, and taking into account the SiO_2_ substrate and hBN capping layer (see Methods). For direct comparison with the experiment, the calculated Kerr signal is assumed to be proportional to ${\sum }_{{{{\rm{z}}}}}\Delta {m}_{{{{\rm{z}}}}}\exp ({{{\rm{-z}}}}/{\lambda }_{p})$^29–31^, where *Δm*_z_ is the laser-induced change of the out-of-plane magnetization, z is the depth within the sample (z = 0 being the surface), and *λ*_*p*_ is the penetration depth of the laser pulse (see Supplementary Section S5). The thickness-dependent remagnetization in the simulations (Fig. 2b) closely follows the trend observed in the experiments. Based on the data obtained at long time delays, 30 nm is identified as a transition thickness, where the remagnetization begins at time delays slightly below 3000 ps. The fluence-dependent response of the 30 nm thick flake obtained from the experiment and simulation is shown in Fig. 2c–d, respectively. At fluence *F* = 0.15 mJ/cm^2^ around 20% demagnetization is achieved, followed by gradual remagnetization. Similar behavior is observed for other thicknesses (Supplementary Fig. 10). With increasing fluence, the demagnetization amplitude increases and its peak value shifts towards shorter time delays (<500 ps), in agreement with previous theoretical^13^ and experimental^27^ studies of bulk CGT. This behavior is associated with a two-step demagnetization process^19^ due to a relatively weak electron-spin coupling in the CGT, which is common to other 2D vdW magnets such as Fe_3_GeTe_2_^32^ and CrI_3_^11,16^. As the fluence increases, the second step of the demagnetization process gradually disappears and the demagnetization process evolves from type-II to type-I, which in consequence shifts the demagnetization peak towards shorter time delays. Full demagnetization is achieved at *F* = 0.67 mJ/cm^2^ while the further increase of the fluence allows the full demagnetization to be achieved at shorter time delays but slows down the remagnetization (Fig. 2c). The experimental results are qualitatively reproduced by theory (Fig. 2d), where quantitative discrepancies are likely due to a mismatch in fluence values and uncertainty about the temperature-dependent heat capacity of the substrate. Furthermore, the calculations consider only the heat transport along the out-of-plane direction. This simplification is justified since the thickness of the film is much smaller than both its lateral extent and the laser spot size, and so the heat provided by the laser pulse diffuses primarily in the out-of-plane direction before dissipating within the substrate. Nevertheless, lateral transport may lead to greater non-uniformity of the temperature profile within the substrate in the case of the experiment and increase the rate at which heat is removed from the pumped region of the film. This would have the effect of reducing the demagnetization achieved in the experiment.

**Fig. 3:**
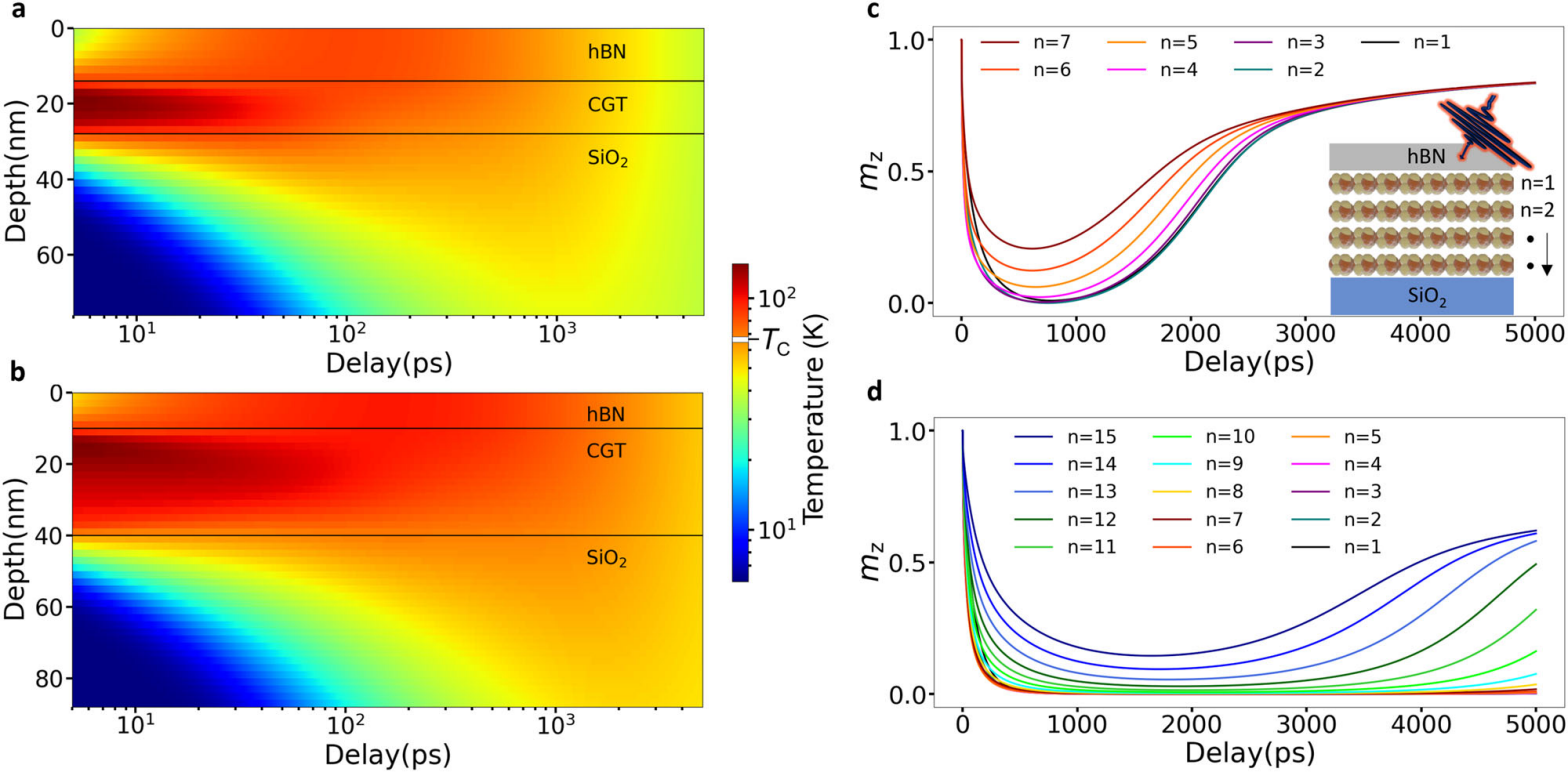
Depth profiles of laser-induced changes of phonon temperature and magnetization from 3TM simulations. **a, b** Phonon temperature maps across the hBN/CGT/SiO_2_ with CGT thickness of 14 nm and 30 nm, respectively. **c, d** Normalized magnetization *m*_z_ component for each CGT layer *n* = 1 − 7 (**c**) and *n* = 1 − 15 (**d**) for 14 nm and 30 nm thick CGT, respectively. The laser fluence was *F* = 0.5 mJ/cm^2^. The hBN thickness is 14 nm (**a, c**) and 10 nm (**b, d**). In all cases the SiO_2_ is 300 nm thick.

The combined set of experimental and theoretical results demonstrate thickness-dependent demagnetization and remagnetization in a 2D vdW magnet. Generally, phonons are the main heat carriers in semiconductors and most vdW materials, thus the phonon temperature^13,28^ provides more insight into the heat dissipation through the CGT flake down into the underlying substrate SiO_2_. In the present case, the SiO_2_ layer is 300 nm thick in all the samples studied, while the presence of the Si substrate underneath is neglected in further discussion. In Fig. 3a, b, the laser-induced time-dependent change in phonon temperature is plotted in the out-of-plane direction (depth of the sample) for 14 nm and 30 nm thick CGT flakes, respectively (see Supplementary Fig. 13 for other CGT thicknesses). Apart from the interfaces, the temperature appears to be more or less uniform across the 14 nm thick CGT, while a temperature gradient is developed along the depth of the 30 nm flake. This is due to the finite penetration of the pump beam (*λ*_*p*_ ~ 32 nm), which causes heat to be deposited primarily within the upper CGT layers, making the temperature gradient more pronounced for CGT thicknesses comparable to or larger than the penetration depth. Due to the poor thermal conductivity of the CGT along the out-of-plane direction (*κ* = 1 W/mK)^33^, the phonon temperature in the 30 nm thick CGT remains above the Curie temperature for several nanoseconds after the excitation (Fig. 3b). By reducing the thickness of the CGT, the distance from the hottest region of the CGT to the adjacent SiO_2_ layer is also reduced. The SiO_2_ acts as a heat sink layer and allows the phonon temperature to fall on shorter timescales. Regardless of the thickness of the CGT, a temperature gradient is observed at the CGT/hBN interface at time delays below 100 ps. The thermal conductivity of the hBN (*κ* = 5 W/mK)^34,35^ is much larger than that of CGT, but the heat can be efficiently transferred only from the interfacial CGT layers.

The observed changes in phonon temperature are reflected in the dynamics of remagnetization, as illustrated in the layer-resolved depth profile of the magnetization *m*_z_ vs time delay in Fig. 3c, d for 14 nm and 30 nm thick CGT, respectively. For the 14 nm case, full demagnetization is achieved at around 700 ps and can only be observed in the top four layers (note that here each layer corresponds to 2 nm of CGT). All layers follow similar magnetization dynamics, start to remagnetize after 800 ps, and converge to the same value at around 3000 ps. For 30 nm thick CGT, all but the four bottom layers (*n* = 12 – 15) achieve full demagnetization. The remagnetization is much slower as compared to the case of the 14 nm thick sample, and the top layers (*n* = 1 – 7) show no signs of recovery even after 5000 ps delay. The difference in remagnetization behavior of thin and thick CGT samples can be understood in terms of the amount of heat remaining in the system with increasing time (Fig. 3c–d). It takes longer for heat to be transported from within the thicker CGT layers to the substrate, and so they remagnetize more slowly. Note that modest differences in the hBN thickness, such as between 14 nm and 10 nm used in Fig. 3a, c and Fig. 3b, d to match the experiment, have a negligible effect on the demagnetization and remagnetization process of the CGT (Supplementary Fig. 15).

Furthermore, we demonstrate that the ultrafast spin dynamics can be also controlled via the thermal conductivity of the underlying substrate. By extending the 3TM simulations to other potential substrate materials (Al_2_O_3_, MoSe_2_, Bi_2_Te_3_, ZnO, black phosphorus, AIN), we observed that both the demagnetization and remagnetization timescales are highly sensitive to the evolution of the phonon temperature within the substrate, which dictates the heat conduction across the interface (Supplementary Fig. S24). This behavior is largely determined by the thermal conductivity and reveals the possibility of controlling magnetization dynamics via a non-magnetic parameter. Finally, it is worth nothing that non-equilibrium effects due to the non-thermal nature of the spin excitations associated with the ultrafast demagnetization and remagnetization processes are also present, as thoroughly discussed in Supplementary Section S10.

So far, the discussion has focused on bypassing the low thermal conductivity and slow heat conduction, in order to achieve more efficient remagnetization. However, in certain applications such as spin caloritronics^36^, low thermal conductivity might be desirable. Laser-induced magnetization processes such as nucleation and manipulation of topological spin textures^13,37^ have attracted considerable attention. Such phenomena often require high laser fluence and the absence of external fields, resulting in random reorientation of magnetic domains after each laser pulse^38^. This makes real-space time-resolved imaging challenging since stroboscopic measurements will average out the stochastic signal accumulated after each pulse^39,40^.

**Fig. 4:**
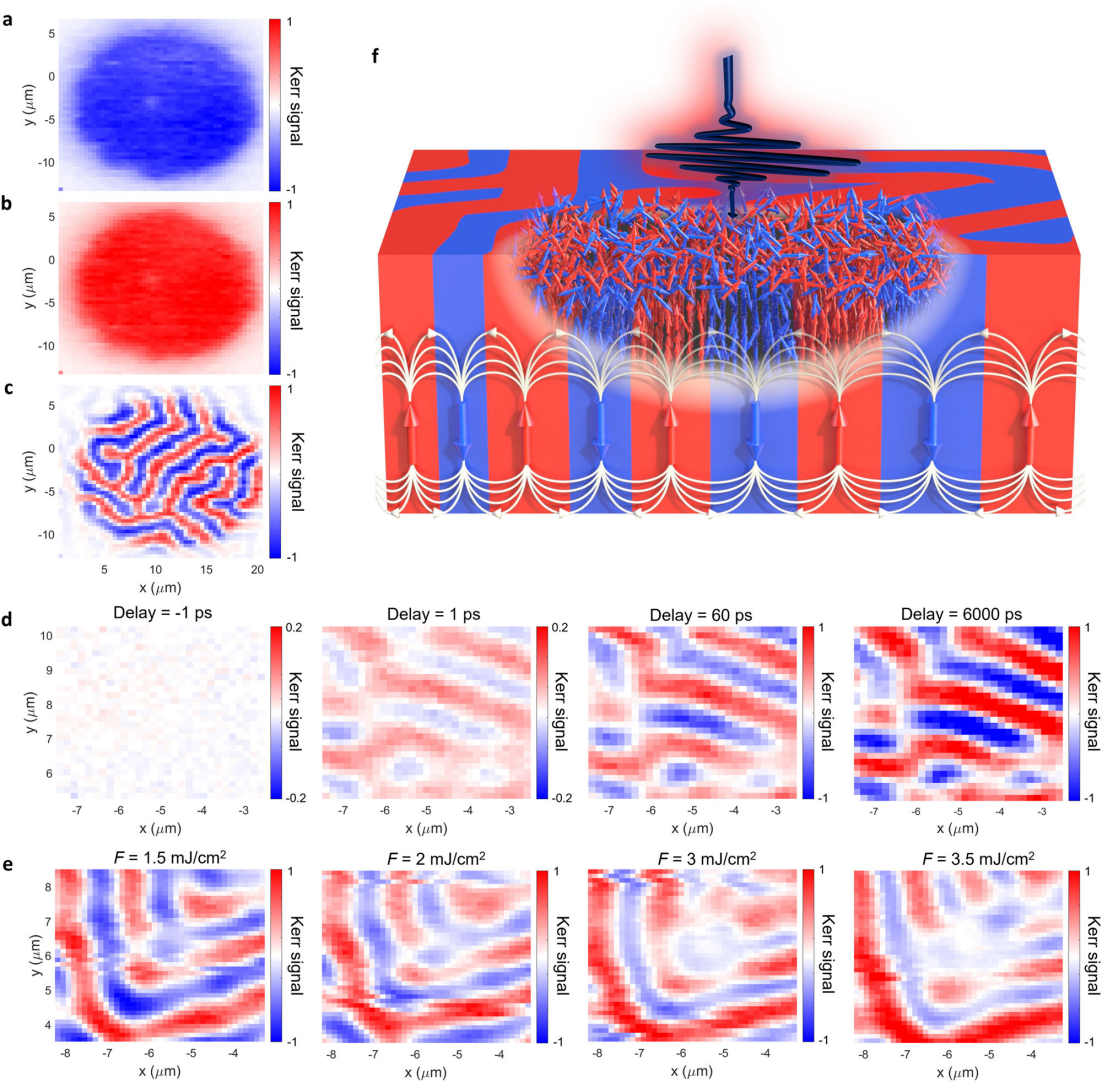
Magnetic domain memory effect. **a**–**c** Time-resolved MOKE images of the demagnetization of the domain structure at 1000 ps delay and for *F* = 1 mJ/cm^2^ with magnetic field *μ*_0_*H* = - 0.3 T (**a**), + 0.3 T (**b**) and 0 T (**c**), respectively. **d** Time-resolved MOKE images at *μ*_0_*H* = 0 T and *F* = 1 mJ/cm^2^ for different time delays. **e** Time-resolved MOKE images at *μ*_0_***H*** = 0 T and at 1000 ps delay for different fluences *F*. **f** Schematic representation of the demagnetization process. Due to the finite penetration depth of the pump laser, the bottom layers stay intact during excitation and can imprint the domain structure pattern into the top layers spin via their stray field (white lines) as the magnetization recovers.

Here it is demonstrated that in the case of thick CGT, the magnetic domain pattern at remanence remains intact even during the process of full demagnetization, keeping in mind that the penetration depth of the optical pump will limit the volume of the demagnetized region, and the penetration of the optical probe results in a Kerr signal that is primarily due to the top layers of the crystal. Figure 4a–c show an example of time-resolved imaging at fixed time delay of 1000 ps and fluence *F* = 1 mJ/cm^2^ leading to full demagnetization (which corresponds to  ± 1 change in the normalized Kerr signal). As expected, under the field applied *μ*_0_*H* = - 0.3 T (Fig. 4a) and *μ*_0_*H* = + 0.3 T (Fig. 4b), the demagnetization signal changes polarity because after each pulse the magnetization returns to its equilibrium position and aligns along the field direction. Interestingly, at remanence (*μ*_0_*H* = 0 T), the same change of the Kerr signal is observed within particular domains (Fig. 4c). Therefore, even though the domains become fully demagnetized, they retrieve their exact shape and size after each laser pulse. Otherwise, it would not be possible to see magnetic domains in the stroboscopic measurement. The domain patterns recorded during time-resolved measurements are the same as the static domain patterns, which can be recorded solely with the probe beam (see Supplementary Fig. 16). Regardless of the applied field, in all three cases the Kerr signal decays while moving away from the pump beam position, which further confirms that the observed signal originates from the transient, optically induced changes to the magnetization. This also demonstrates the unique capability of the beam-scanning microscopy to visualize the spatial profile of the ultrafast pump pulse. Figure 4d shows the evolution of the domain pattern as a function of time delay. The demagnetization increases with increasing delay but there are no modifications to the domain pattern configuration. Note that changes on a nanometer scale such as transient domain wall broadening due to superdiffusive spin transport across domain boundaries^41^ are too small to be observed with the optical methods used here.

Finally, it is shown that even at a fluence of *F* = 3.5 mJ/cm^2^, which is more than three times that required for full demagnetization, the domains still retain their shape (Fig. 4e). However, in the centre of the imaged area, a circular spot with weaker intensity starts to appear due to permanent changes to the sample (see Supplementary Fig. 17). Thus, the remanent domain structure is very robust, and can be only destroyed by laser fluences above the damage threshold. Furthermore, the effect persists in the presence of small bias magnetic fields and is observed when starting from different initial domain patterns (Supplementary Figs. 18–19). This proof-of-concept makes CGT, and potentially other 2D vdW magnets with similar thermal properties, an ideal candidate for a magnetic domain memory (MDM). Previously, MDM has been demonstrated in a limited range of fields and temperatures by combining soft and hard magnetic layers^42^ and antiferromagnetic layers^25^ but not in response to laser pulses. The unique ability to preserve the magnetic pattern under the influence of highly intense optical excitation is possible thanks to the low thermal conductivity. The most plausible explanation is that the magnetic moments lying well below the penetration depth of the optical pump remain intact and restore the magnetic order in the upper, demagnetized layers, via stray field (as shown schematically in Fig. 4f). Hence, our results reveal a mechanism for temporal control of the magnetic state that preserves topology during the demagnetization and remagnetization process. Regardless of the initial arrangement of magnetic domains, which for the CGT can also be set by applying intense laser pulses^13^, the optically-induced transient changes to the magnetization will always exhibit the same spatial pattern (unless large fluences are used as in^13^). An example of the transient evolution of the domain structure, starting from a state in which stripe domains and bubbles/skyrmions coexist, is shown in Supplementary Fig. 19.

## Outlook

The results presented here demonstrate how the temperature of phonons and spins in a 2D van der Waals magnet can be modified on picosecond timescales, which will be of crucial importance for applications in nanoscale spintronics and spin caloritronics. Using time-resolved MOKE imaging in combination with microscopic spin modeling, it has been shown that remagnetization following laser-induced demagnetization can be substantially accelerated by reducing the thickness of the 2D magnet. We reveal that a universal picture of how heat affects the ultrafast spin dynamics can be drawn in terms of the thermal conductivity of a 2D magnet and the underlying substrate. The measurement techniques used in our study can be promptly extended to other classes of vdW magnets with a small optically induced modulation of the magnetization. This can be used to image domain structure with increased sensitivity. The robust magnetic domain memory effect observed in our results motivates further studies of topological switching, time-resolved imaging and non-volatile memory in other systems with low thermal conductivity. In summary the reduced dimensionality of 2D vdW magnets can be exploited for meaningful control of spin dynamics that is unreachable in other materials.

## Methods

### Sample preparation

Single-crystalline Cr_2_Ge_2_Te_6_ (CGT) were synthesized using the chemical vapor transport method, described in detail in^13^. High-quality hBN crystals were purchased from HQ Graphene. Si/SiO_2_ (300 nm) substrates were prepared through a standard cleaning procedure to enhance flake adhesion and promote exfoliation of thin CGT flakes. The substrates were cleaned with Acetone and IPA to remove surface contamination and subsequently underwent plasma treatment (5 minutes of 5 W plasma utilizing a gas mixture of Argon and Oxygen). Finally the substrates were heated for 2 minutes at 333 K on a hot plate to further enhances the adhesion. Bulk CGT crystals were cleaved into thin laminae using Blue Nitto tape in a nitrogen-filled glove box to protect from oxidation and moisture (O_2_  < 1 ppm; H_2_O  < 0.02 ppm). The tape was then brought into contact with the prepared substrate and slowly peeled off at a low angle for optimized flake sizes. After suitable flakes were localized through optical contrast, thin hBN capping layer were transferred on top of the CGT flakes via the PPC method using HQ Graphene transfer station. To determine flake thickness, Atomic Force Microscopy (AFM) was conducted on the encapsulated flakes using Bruker ICON in a tapping mode. Gwyddion software was used for AFM data analysis.

### Time-resolved beam-scanning MOKE microscopy

Laser pulses produced by a Monaco 1035 fiber laser (Coherent, 1035 nm fundamental) seeds an optical parametric amplifier (OPA) (Opera-F, Coherent) with a signal output wavelength of 650 - 900 nm and an average pulse width of 25 fs. The 800 nm output of the OPA is used as the pump beam, while the 400 nm probe is obtained via a second harmonic generation (SHG) process. The pump passes through a mechanical delay stage and its path is stabilized with additional beam stabilization optics (MRC systems). The pump (800 nm) and probe (400 nm) beams are combined with a dichroic mirror and directed into a microscope objective, which focuses the probe beam to a diffraction-limited spot size. The pump beam with linear polarization, 50 fs pulse duration, and 1 MHz repetition rate is incident at normal incidence and focused to either a 50 *μ*m or 200 *μ*m diameter (intensity at 1/*e*^2^) spot, by a  × 20 and  × 5 objective lens, respectively. The pump pulse train was modulated at 891 Hz using a mechanical chopper. Modulated, pump-induced changes in the probe beam polarization and reflectivity are detected by a lock-in amplifier (LIA) at the chopper modulation frequency. A dual-axis galvo mirror scanning system (Thorlabs) is used to scan the position of the probe beam on the sample. Although the probe scanning mechanism alters the path of the probe beam, the geometry of the system is such that the path length introduced by tilting the first mirror is compensated with the second mirror, and as a result, there is very little variation in the pump-probe delay as the probe beam is moved relative to the pump, as verified in a similar setup in^43^. Note also that any depolarization of the probe beam acquired due to the tilt is automatically removed in time-resolved measurements since the LIA only detects changes to the polarization induced by the pump beam at the reference frequency. The measurements were performed in an LHe-flow MicrostatMO (Oxford Instruments) at a base temperature of 6 K and with the magnetic field applied perpendicular to the sample plane.

### Wide-field Kerr microscopy (WFKM)

The polar Kerr effect was used to sense the out-of-plane magnetization in response to a magnetic field. The sample illumination was linearly polarized, while polarization changes of the reflected light due to the polar Kerr effect were detected as intensity changes using a nearly crossed analyzer, quarter-waveplate, and high-sensitivity CMOS camera. Measurements were performed at 12 K.

### Microscopic three-temperature model

The ultrafast magnetization dynamics, as explained by the three-temperature model (3TM), consider the intrinsic mechanism of electron-phonon-mediated spin flips^19^. In this framework, the model describes the energy of internally thermalized electron and lattice systems through their respective temperatures, *T*_e_ and *T*_p_, and their corresponding specific heats, *C*_e_ and *C*_p_. The 3TM assumes that the spin system remains non-internally thermalized, so it is not possible to describe the magnetization changes using a single temperature. Instead, the microscopic portrayal involves spin-flips triggered by electron-phonon scattering events. These intricate processes give rise to net magnetization dynamics, and we can understand them by combining the two-temperature model and an equation of motion for the non-equilibrium macroscopic magnetization distribution.

### Energy dynamics

3TM is described by two coupled equations of motion for the electron and phonon temperatures:

1 $${C}_{{{{\rm{e}}}}}\frac{d{T}_{{{{\rm{e}}}}}}{dt}=\frac{\partial }{\partial z}\left({k}_{e}\left(\frac{{T}_{e}}{{T}_{p}}\right)\frac{\partial {T}_{e}}{\partial z}\right)+{g}_{{{{\rm{e-p}}}}}({T}_{{{{\rm{p}}}}}-{T}_{{{{\rm{e}}}}})+S(z,t)+{\dot{Q}}_{{{{\rm{es}}}}}$$

2 $${C}_{{{{\rm{p}}}}}\frac{d{T}_{{{{\rm{p}}}}}}{dt}=\frac{\partial }{\partial z}\left({k}_{p}\frac{\partial {T}_{p}}{\partial z}\right)-{g}_{{{{\rm{e-p}}}}}({T}_{{{{\rm{p}}}}}-{T}_{{{{\rm{e}}}}})$$

The absorbed laser pulse *S*(*t*) instantaneously excites the electron and is subsequently thermalized, followed by scattering with the phonon subsystem. The electron heat capacity is *C*_e_ = *γ*_e_*T*_e_. *k*_*e*_ and *k*_*p*_ are the out-of-plane electronic and phononic thermal conductivities. The electron-phonon coupling *g*_e-p_ enables temperature equalization between hot electrons and the lattice. This process occurs within a timeframe determined by the ratio of *g*_e-p_/*C*_e_. ${\dot{Q}}_{{{{\rm{e-s}}}}}$ defines the energy exchange between the spin and electron systems. A crucial element of the two-temperature model in Eqs. (1) and (2) lies in the precise determination of input parameters such as *C*_e_, *C*_p_, *k*_*e*_, and *k*_*p*_. We source these parameters from experimental data, conveniently presented in Table 1.

**Table 1:** Input parameters for the 3TM used on the ultrafast laser-induced magnetic dynamics on CGT

Quantity	Description	hBN	CGT	SiO_2_	Units
*γ*_*e*_	Sommerfeld constant	0.0	736.87^28^	0.0	J/m^3^K^2^
*C*_*p∞*_	maximum lattice spec. heat	2.645 × 10^6^	1.38 × 10^6^^46^	1.9 × 10^6^	J/m^3^K
*g*_*e*−*p*_	el.-ph. coupling	0.0	15 × 10^16^^28^	0.0	W/m^3^K
*k*_*e*_	el. conductivity	0.0	0.0016	0.0	W/mK
*k*_*p*_	ph. conductivity	5.0^34,35^	1.0^33^	1.5^47,48^	W/mK
*T*_*C*_	Curie temp.	0.0	65^46^	0.0	K
*T*_*Debye*_	Debye temp.	400	200^46^	403	K
*T*_*Ein*_	Einstein temp.	300	131	302.25	K
S	effective spin	0	3/2^46^	0	
*μ*_*at*_	atom. magn. moment	0.0	4	0.0	*μ*_*B*_
*a*_*sf*_	spin-flip prob.	0.0	0.05	0.0	
*V*_*at*_	atomic volume	100	100	100	Å^3^

*γ*_e_ and *g*_e-p_ of CGT were obtained from^28^. *C*_p∞_, *T*_C_, *T*_Debye_, and S of CGT were obtained from^46^.

The pump pulse is assumed to have Gaussian temporal profile centered at *t*_0_ with duration *σ*, and to follow Lambert-Beer absorption along the z-axis,

3 $$S(z,t)=\frac{{S}_{0}}{\lambda }\,{e}^{-\frac{{(t-{t}_{0})}^{2}}{2{\sigma }^{2}}}\,{e}^{-z/{\lambda }_{p}}.$$

*S*_0_ represents the energy density from the laser, which is absorbed by the electron system. *λ*_*p*_ is the penetration depth as defined in the text. When the energy input from the laser pump ceases, this surplus energy is transferred to both (i) the lattice by *g*_e-p_(*T*_e_ − *T*_p_) and (ii) the spin system via^44^,

4 $${\dot{Q}}_{{{{\rm{e-s}}}}}=Jm\dot{m}/{V}_{{{{\rm{at}}}}}.$$

Here *V*_at_ is the mean atomic volume, calculated by dividing the unit cell volume by the number of atoms in a unit cell and the exchange energy *J* is linked to the Curie temperature through the mean field approximation (MFA) by

5 $$J=3\frac{{S}^{2}}{S(S+1)}{k}_{B}{T}_{C}.$$

The specific heat of the electron system is computed in the Sommerfeld (free electron model) approximation

6 $${C}_{{{{\rm{e}}}}}={\gamma }_{{{{\rm{e}}}}}{T}_{{{{\rm{e}}}}},$$

where *γ*_e_ is the Sommerfeld coefficient and the lattice specific heat is computed with the Einstein model, where

7 $${C}_{{{{\rm{p}}}}}={C}_{{{{\rm{p}}}}\infty }\frac{{T}_{{{{\rm{Ein}}}}}^{2}}{{T}_{{{{\rm{p}}}}}^{2}}\frac{\exp \frac{{T}_{{{{\rm{Ein}}}}}}{{T}_{{{{\rm{p}}}}}}}{{(\exp \frac{{T}_{{{{\rm{Ein}}}}}}{{T}_{{{{\rm{p}}}}}}-1)}^{2}}.$$

We use experimentally measured Debye temperature as an input for our model through the relation *T*_Ein_ ≈ 0.75 *T*_Debye_.

### Magnetisation dynamics equations

The magnetization dynamics for arbitrary value of spin *S* can be described by the statistical change of occupation numbers ${f}_{{m}_{s}}$ of the *S*_*z*_ component *m*_*s*_:

8 $$\frac{dm}{dt}=-\frac{1}{S}{\sum }_{ms=-S}^{ms=+ S}{m}_{s}\frac{d{f}_{{m}_{s}}}{dt}$$

9 $$\frac{d{f}_{{m}_{s}}}{dt}=-({W}_{{m}_{s}}^{+}+{W}_{{m}_{s}}^{-}){f}_{{m}_{s}}+{W}_{{m}_{s-1}}^{+}{f}_{{m}_{s-1}}+{W}_{{m}_{s+1}}^{-}{f}_{{m}_{s+1}}$$

10 $${W}_{{m}_{s}}^{\pm }=R\frac{Jm}{4S{k}_{B}{T}_{c}}\frac{{T}_{p}}{{T}_{c}}\frac{{{\mbox{e}}}^{\mp \frac{Jm}{2S{k}_{B}{T}_{e}}}}{{\mbox{sinh}}\,\left(\frac{Jm}{2S{k}_{B}{T}_{e}}\right)}(S(S+1)-{m}_{s}({m}_{s}\pm 1))$$

The rate parameter *R* depends on the microscopic parameters of the system, and it is proportional to the spin-flip probability, *a*_sf_.

11 $$R=8\frac{{a}_{{{{\rm{sf}}}}}{g}_{{{{\rm{e-p}}}}}{T}_{C}^{2}{V}_{{{{\rm{at}}}}}}{{\mu }_{{{{\rm{at}}}}}{k}_{B}{T}_{{{{\rm{Debye}}}}}^{2}},$$

where *μ*_at_ is the atomic magnetic moment in units of the Bohr magneton and *k*_*B*_ is the Boltzmann constant.

The first term in Eq. (9) describes the reduction of occupation through scattering into higher and lower neighboring spin-levels. The increase of occupation of higher and lower spin-levels is described respectively by the second and third terms. The analytical description of the transition rate ${W}_{{m}_{s}}^{\pm }$ was introduced by Beens et al.^45^ using Fermi’s golden rule to describe the spin-flip process associated with the electron-phonon scattering, beginning from the Hamiltonian

12 $${{{{\mathcal{H}}}}}_{{{{\rm{eps}}}}}= \sqrt{\frac{{a}_{sf}}{{D}_{S}}}\frac{{\lambda }_{ep}}{{N}^{3/2}}{\sum}_{k,{k}^{{\prime} }}{\sum }_{q}^{N{D}_{P}}{\sum }_{j}^{{N}_{S}}{c}_{k}^{{{\dagger}} }{c}_{{k}^{\prime} }({s}_{j,+}+{s}_{j,-})({a}_{q}^{{{\dagger}} }+{a}_{q})$$

where ${a}_{q}^{{{\dagger}} }$ and *a*_*q*_ describe the emission and absorption of a phonon respectively.

### Simulation setup: input parameters

The input parameters we have used for our simulations are shown in Table 1. The maximum lattice heat capacity of CGT was extracted from Fig. 2(b) at Debye temperature *T*_*D*_ = 200*K* of reference^46^, then converted to unit *J*/*m*^3^*K* by dividing the molar volume (1.67 × 10^−4^*m*^3^/*mol*). The Sommerfeld constant and electron-phonon coupling parameter of CGT were obtained from reference^28^. The phononic thermal conductivity of CGT, hBN, and SiO_2_ was obtained from^33–35,47,48^. Moreover, in our 3TM approach we take into account the thermal boundary conductance (TBC) (the inverse of thermal boundary resistance)^49^ established at the interface between the CGT and different substrates. The TBC is estimated by averaging the thermal conductivity at the interface and dividing by the cell dimension *L* across the interface^49,50^: (*k*_*CGT*_ + *k*_*substrate*_)/(2 × *L*). The value of *L* was optimized in our simulations resulting in *L* = 2 nm. This led to values of TBC in the range of 500  − 2375 MW m^2^K^−1^ as described in Table 2. These estimates define the upper limit of the TBC values and do not take into account additional contributions such as mismatch in the acoustic impedance and phonon density of states between the two sides of the interface, that inhibit phonon transmission across the interface^49^. The good agreement between measurements and simulations for the CGT/SiO_2_ suggests that our assumptions are sufficient, indicating a high-quality interface that supports efficient heat dissipation. Otherwise, the spin dynamics would not be significantly affected by contact with the SiO_2_ substrate, which has thermal conductivity just 50% larger than the CGT (Table 1).

**Table 2:** Thermal boundary conductance (TBC) calculated for CGT interfaced with different substrates as described in the text with the respective thermal conductivity coefficient (*k*_*p*_) for each substrate^46,49–56^

Interface	TBC (MW m^2^K^−1^)	*k*_*p*_ (W m^−1^K^−1^)
CGT/Al_2_O_3_	500	1.0
CGT/MoSe_2_	550	1.2
CGT/SiO_2_	650	1.5
CGT/Bi_2_Te_3_	700	1.8
CGT/ZnO	825	2.3
CGT/Black Phosphorous	1875	6.5
CGT/AIN	2374	8.5

## Supplementary information

Supplementary Information

Transparent Peer Review file

## Data Availability

All the data supporting the findings of this study are available within the paper, the Supplementary Information, and have been deposited in Open Research Exeter (ORE) repository at 10.24378/exe.5649.
