# Supplementary material for: Ultrafast thermo-optical control of spins in a 2D van der Waals semiconductor
Source: Nat Commun. 2025 Mar 21;16:2797. doi: 10.1038/s41467-025-58065-1 (PMC11928556; doi:10.1038/s41467-025-58065-1)
Supplement: Supplementary file 1 — Supplementary Information [file 41467_2025_58065_MOESM1_ESM.pdf]

Supplementary Information for:

Ultrafast thermo-optical control of spins in a 2D van der Waals semiconductor

Maciej Dąbrowski[1†], Sumit Haldar[2], Safe Khan[3], Paul S. Keatley[1], Dimitros Sagkovits[3], Zekun Xue[3], Charlie Freeman[3], Ivan Verzhbitskiy[4], Theodor Griepe[5], Unai Atxitia[5], Goki Eda[4,6,7], Hidekazu Kurebayashi[3,8,9], Elton J. G. Santos[2,10,11†], Robert J. Hicken[1]

[1]*Department of Physics and Astronomy, University of Exeter, EX4 4QL, United Kingdom*

[2]*Institute for Condensed Matter Physics and Complex Systems, School of Physics and Astronomy, The University of Edinburgh, EH9 3FD, United Kingdom*

[3]*London Centre for Nanotechnology, University College London, 17-19 Gordon Street, London, WCH1 0AH, UK*

[4]*Department of Physics, National University of Singapore, 2 Science Drive 3, Singapore, 117542, Singapore*

[5]*Instituto de Ciencia de Materiales de Madrid, CSIC, Cantoblanco, 28049 Madrid, Spain*

[6]*Centre for Advanced 2D Materials, National University of Singapore, 6 Science Drive 2, Singapore, 117546, Singapore*

[7]*Department of Chemistry, National University of Singapore, 3 Science Drive 3, Singapore, 117543, Singapore*

[8]*Department of Electronic & Electrical Engineering, UCL, London WC1E 7JE, United Kingdom*

[9]*WPI Advanced Institute for Materials Research, Tohoku University, 2-1-1, Katahira, Sendai 980-8577, Japan*

[10] *Higgs Centre for Theoretical Physics, University of Edinburgh, Edinburgh, UK*

[11] *Donostia International Physics Center (DIPC), 20018 Donostia-San Sebastián, Basque Country, Spain*

†Corresponding authors: m.k.dabrowski@exeter.ac.uk, esantos@ed.ac.uk,

This Supplementary Information file includes:

1. Time-resolved beam-scanning MOKE microscopy (Figures 1 - 2)

2. Atomic-force microscopy (AFM) images (Figures 3 - 5)

3. Hysteresis loops and static domain structure (Figures 6 - 7)

4. Additional time-resolved measurements on the CGT flakes (Figures 8 - 10)

5. The penetration depth and Kerr signal (Figure 11)

6. Electron temperature maps and additional phonon temperature maps (Figures 12 - 13)

7. Additional data for layer-resolved magnetization dynamics from simulations (Figure 14)

8. Effect of the hBN thickness on the simulated magnetization dynamics (Figure 15)

9. Additional time-resolved measurements on the CGT crystal (Figures 16 - 19)

10. Non-thermal effects on ultrafast spin dynamics in CGT (Figures 20 - 23)

11. Ultrafast control of spin dynamics via thermal conductivity of a substrate (Figure 24)

12. Supplementary references

1 Time-resolved beam-scanning MOKE microscopy

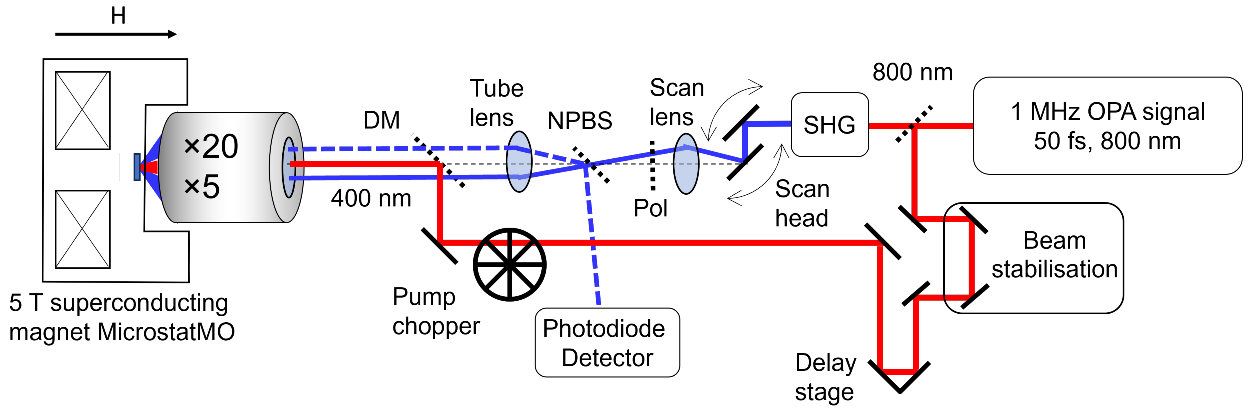

Figure 1: Experimental setup of the time-resolved beam-scanning MOKE microscopy. Laser pulses produced by a Monaco 1035 fibre laser (Coherent, 1035 nm fundamental) seeds an optical parametric amplifier (OPA) (Opera-F, Coherent) with a signal output wavelength of 800 nm and an average pulse width of 25 fs used as the pump beam, while the 400 nm probe is obtained via a second harmonic generation (SHG) process. After reflection from the sample the probe beam passes through a Non-Polarizing Beamsplitter (NPBS) redirecting the beam towards a balanced photodiode detector, which measures the reflectivity and the Kerr signal. The pump passes through a mechanical delay stage and its path is stabilized with additional beam stabilization optics (MRC systems). The pump (800 nm) and probe (400 nm) beams are combined with a dichroic mirror (DM) and directed into a microscope objective, which focuses the probe beam to a diffraction limited spot size. The pump beam with linear polarization, 50 fs pulse duration and 1 MHz repetition rate is incident at normal incidence and focused by a $\times 20$ or $\times 5$ objective lens. The pump pulse train is modulated using a mechanical chopper. A dual-axis galvo mirror scanning system (Thorlabs) is used to scan the position of the probe beam on the sample. Pump-induced changes in the probe beam are detected by a lock-in amplifier (LIA). The sample is placed within a LHe-flow MicrostatMO (Oxford Instruments) at temperature 6 K and with the magnetic field H applied perpendicular to the sample plane.

Due to the Gaussian intensity distribution of the beam, the strength of the laser excitation is not uniform within the area defined by the $1/e^2$ intensity. This experimental fact is usually

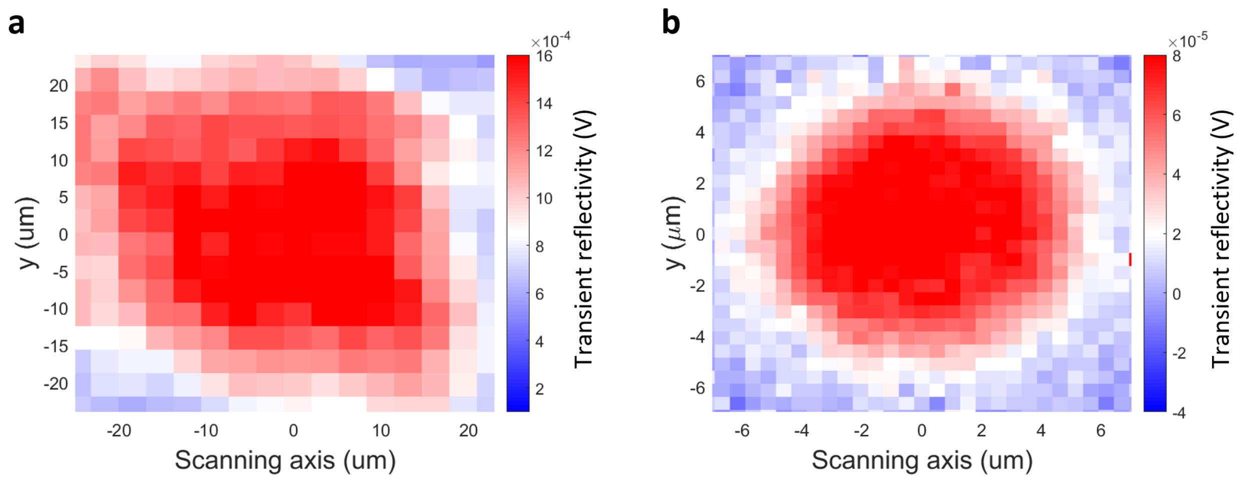

Figure 2: Transient reflectivity at 1 ps time delay acquired via time-resolved beam-scanning microscopy on a reference Co/Pt sample using **(a)** $\times$ 5 and **(b)** $\times$ 20 objective lens. The size of the transient reflectivity beam allows for realistic visualization of the pump beam profile.

neglected in the literature, but is of high importance for spatially resolved studies of thin flakes. The intensity of the excitation was visualized by performing measurements of the transient reflectivity on a reference Co/Pt sample using $\times$ 5 (Fig. 2a) and $\times$ 20 (Fig. 2b) objective lens. For $\times$ 20 lens the transient reflectivity signal is fairly uniform within the area of a circle with 8 μm diameter (Fig. 2b). Hence, we can assume nearly uniform excitation within the 7×7 μm area shown in Figs. 1b-d in the main text. For the objective lens with lower ($\times$ 5) magnification the diameter of the pump beam increases, ensuring homogeneous excitation for the larger area, albeit at the expense of lower spatial resolution achieved with the probe beam. The time-resolved measurements performed with $\times$5 magnification, where the excitation area of the pump is around $\times$ 5 larger as compared to Figs. 1b-d, confirm that the observed differences in the time-resolved Kerr signal are due to the thickness-dependent magnetization dynamics (see Supplementary Fig. 8 for time-resolved images acquired with $\times$5 lens).

2 Atomic-force microscopy (AFM) images

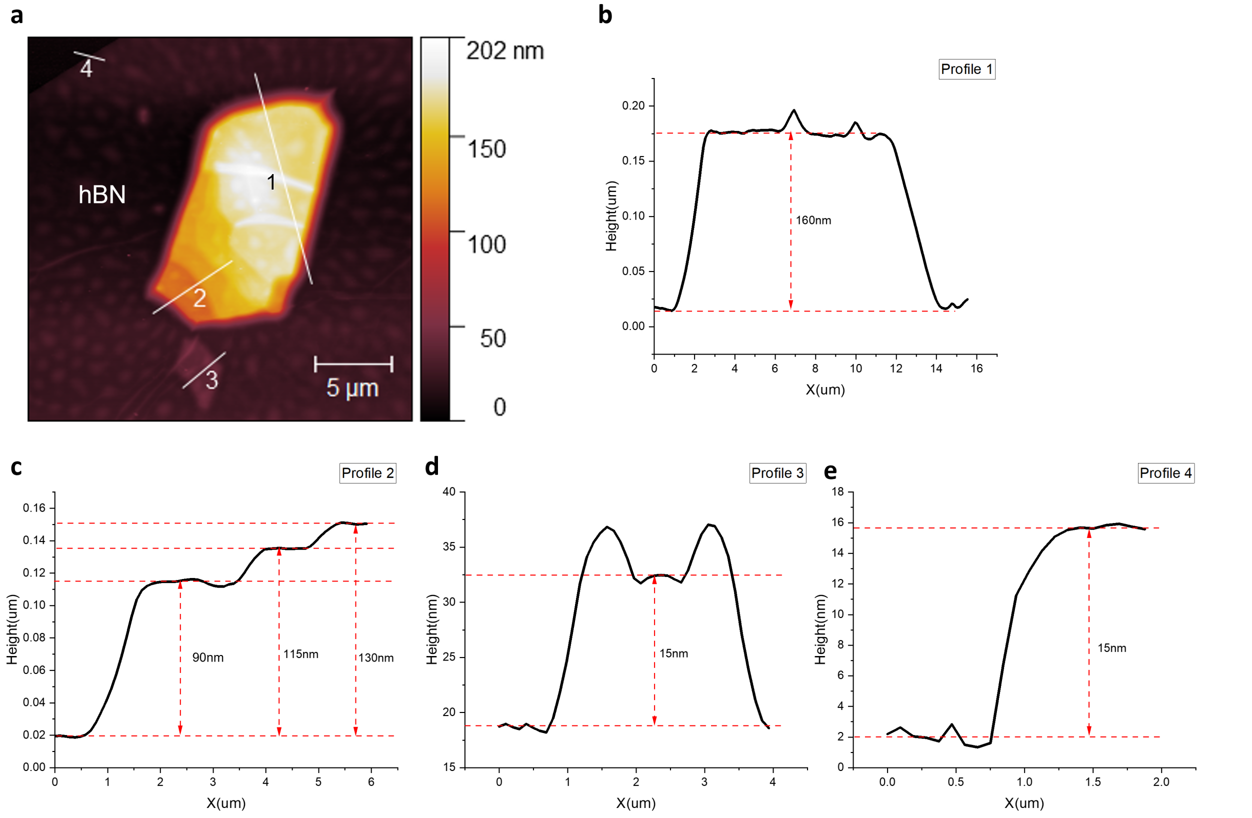

Figure 3: **a,** AFM image of sample 1 consisting of two CGT flakes capped with h-BN and exfoliated on SiO_2/Si. **b, c,** Line profiles 1 and 2 showing various thicknesses of the larger CGT flake. **d,** Line profile 3 for the smaller CGT flake with thickness 15 nm. **e,** Line profile 4 for 15 nm thick hBN.

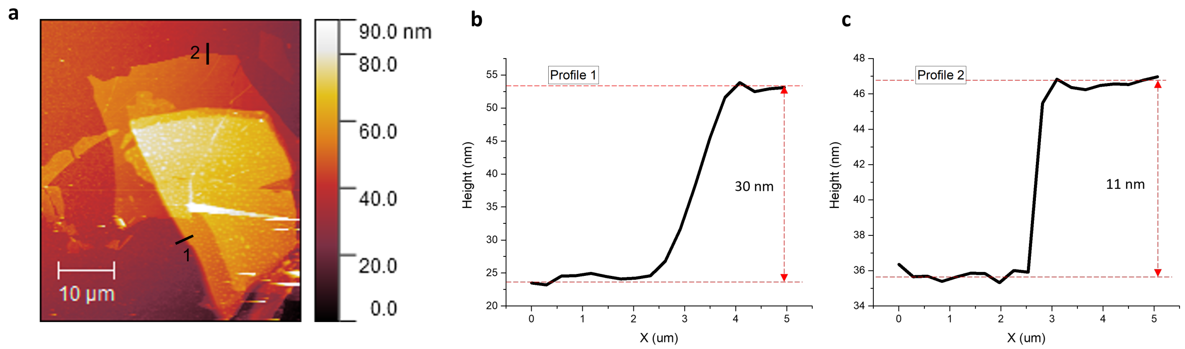

Figure 4: **a,** AFM image of sample 2 with 30 nm thick CGT flake capped with 11 nm thick hBN and exfoliated on SiO_2/Si. **b, c,** Line profiles 1 and 2 for the CGT flake and hBN flake, respectively.

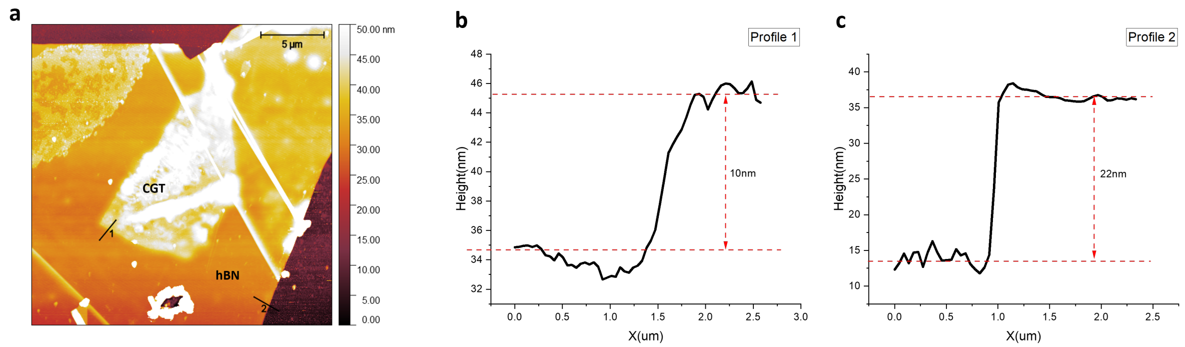

Figure 5: **a,** AFM image of sample 3 with 10 nm thick CGT flake capped with 22 nm thick hBN and exfoliated on SiO_2/Si. **b, c,** Line profiles 1 and 2 for the CGT flake and hBN flake, respectively.

3 Hysteresis loops and static domain structure

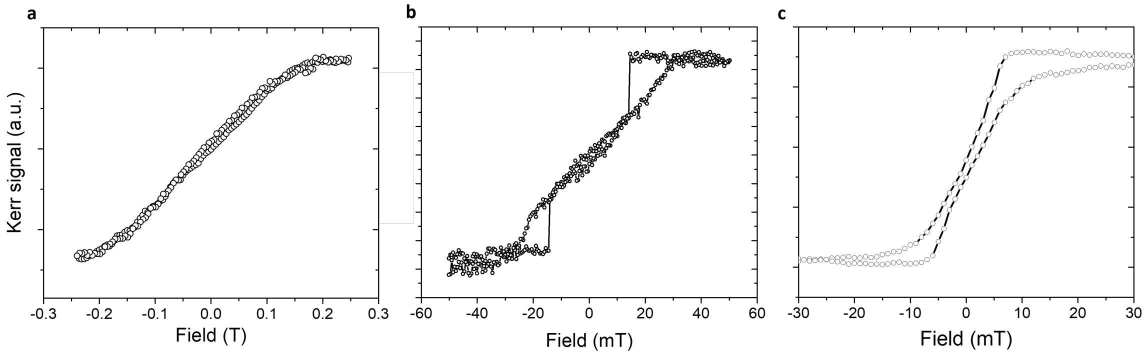

Figure 6: **Hysteresis loops. a,** The CGT crystal (500 μm thick); **b,** Sample 1 (15 nm thick CGT); **c,** Sample 3 (10 nm thick CGT). **(a)** was measured at 6 K using beam-scanning MOKE microscopy while **(b,c)** at 12 K using wide-field Kerr microscopy.

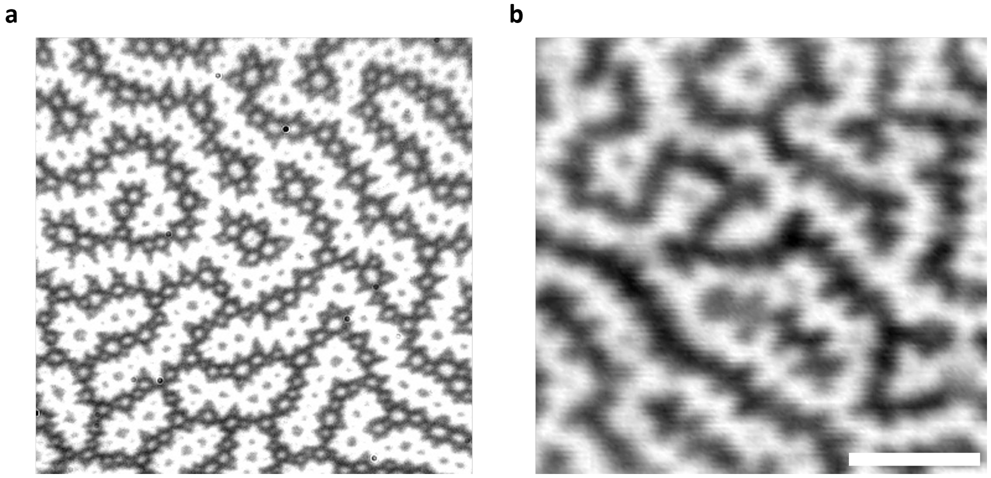

Figure 7: Remanent domain structure of the CGT crystal acquired with: **a,** wide-field Kerr microscopy (WFKM) at T = 12 K using $\times$ 60 objective lens and white LED light source; **b,** beam-scanning MOKE microscopy at T = 6 K using $\times$ 20 objective lens and 400 nm laser light at 1 MHz repetition rate and 50 fs pulse duration. The scale bar has 5 μm length. This comparison demonstrates that the static imaging capability of beam-scanning MOKE microscopy is comparable to commercially available WFKM. The resolution of **(b)** can be further improved by using higher magnification lens and CW laser probe.

4 Additional time-resolved measurements on the CGT flakes

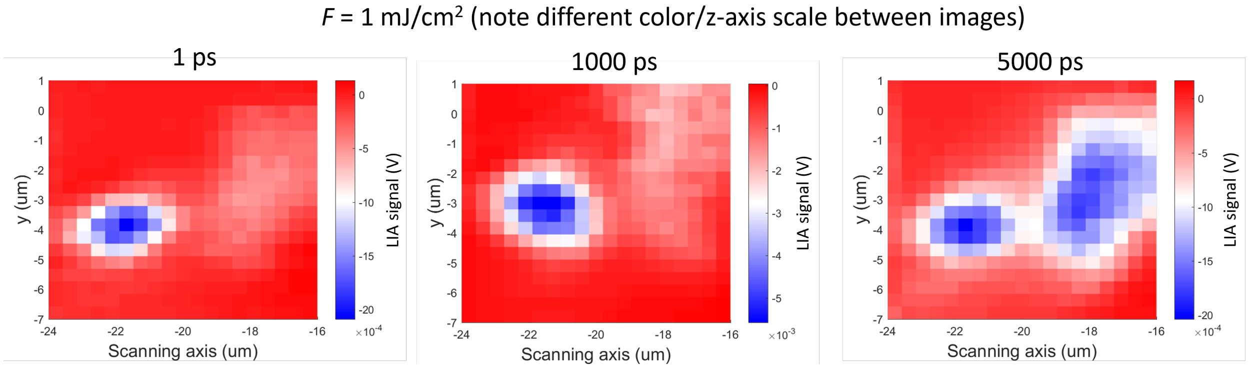

Figure 8: Time-resolved images acquired with $\times 5$ objective lens from the same area of the sample as in Fig.1b-f in the main text. H = -0.5 T, T = 6 K.

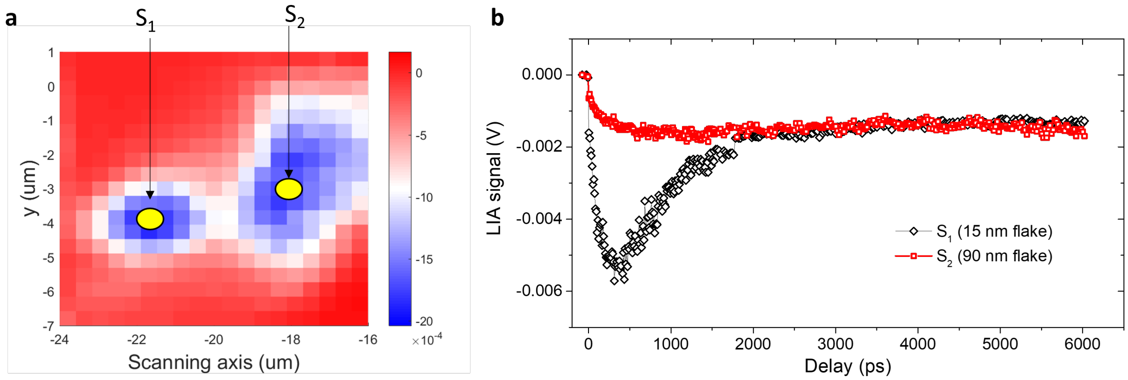

Figure 9: **a,** Time-resolved MOKE image at 5000 ps delay acquired with $\times 5$ objective lens from the same area of the sample as in Fig.1b-f in the main text. **b,** Time-resolved MOKE signal prior to normalization, at fixed position S_1 and S_2 corresponding to 15 nm and 90 nm thick CGT flake. H = -0.5 T, T = 6 K. Although both flakes are fully demagnetized at the fluence used here $F = 1$ mJ/cm^2, the dynamic Kerr signal has different strength for 15 nm and 90 nm flakes due to different, thickness-dependent static Kerr signal for both thicknesses.

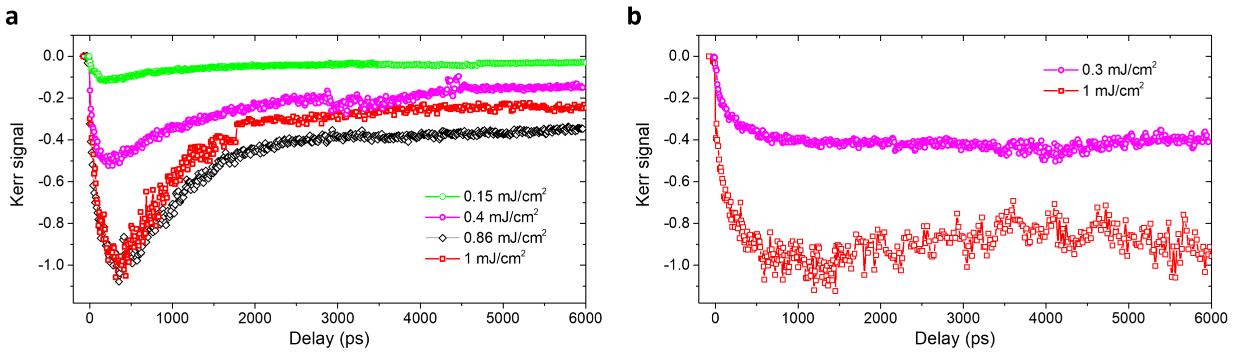

Figure 10: Fluence dependence for 15 nm (**a**) and 90 nm (**b**) thick CGT flakes. The Kerr signal was normalized to the full demagnetization.

5 The penetration depth and Kerr signal

We estimate the penetration depth of the laser based on Abeles' matrix method [7]. In this method, the electric field in +z direction scales with $\exp(\mathrm{i}2\pi\nu n\cos\theta z)$, where n is the complex refractive index, θ is the angle of incidence, ν is the wavenumber of the incident light and z is the z position in the sample. The electric field has another component that travels in the -z direction. The amplitude of that wave is significantly smaller. The absorption scales in intensity, and the intensity scales quadratically in the electric field. The penetration depth can be expressed by $\lambda_p = \frac{1}{2\pi \mathrm{Im(n)}\nu}$. By using refractive indices from [1–3], we obtained λ_p =15.91 nm for the probe (400 nm) and λ_p =31.88 nm for the pump (800 nm) (see Fig. S16).

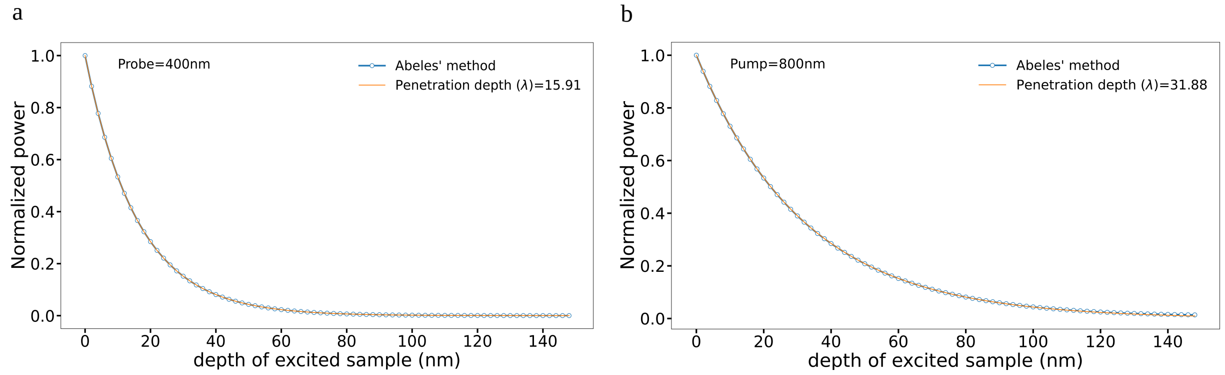

Figure 11: Normalized absorption profile for probe wavelength 400nm (a) and pump wavelength 800nm (b) in 150 nm thick CGT fitted with an exponential function $exp(-z/\lambda_p)$.

In general, the Kerr signal is linearly proportional to the magnetization and, due to the finite penetration of the light, represents a weighted average of the instantaneous magnetization at different depths within the flake [4–6]. More specifically, the Kerr signal measured in the time resolved experiment is proportional to $\sum_z \Delta m_z exp(-z/\lambda_p)$, where Δm_z is the laser-induced change of the

out-of-plane magnetization, z is the depth within the sample ($z = 0$ being the surface), and λ_p is the penetration depth of the laser pulse.

6 Electron temperature maps and additional phonon temperature maps

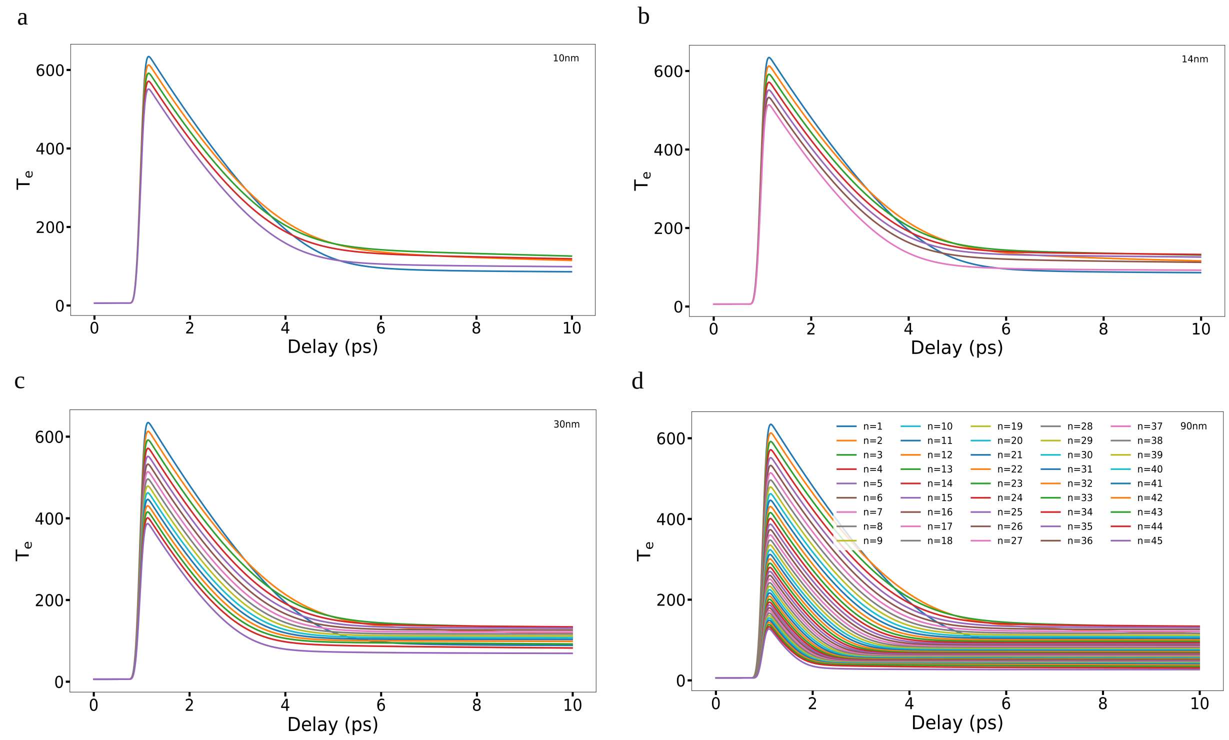

Figure 12: Electron temperatures for 10nm (a), 14nm (b), 30nm (c), and 90nm (d) thick of CGT from simulations. All the layers are shown in different colors.

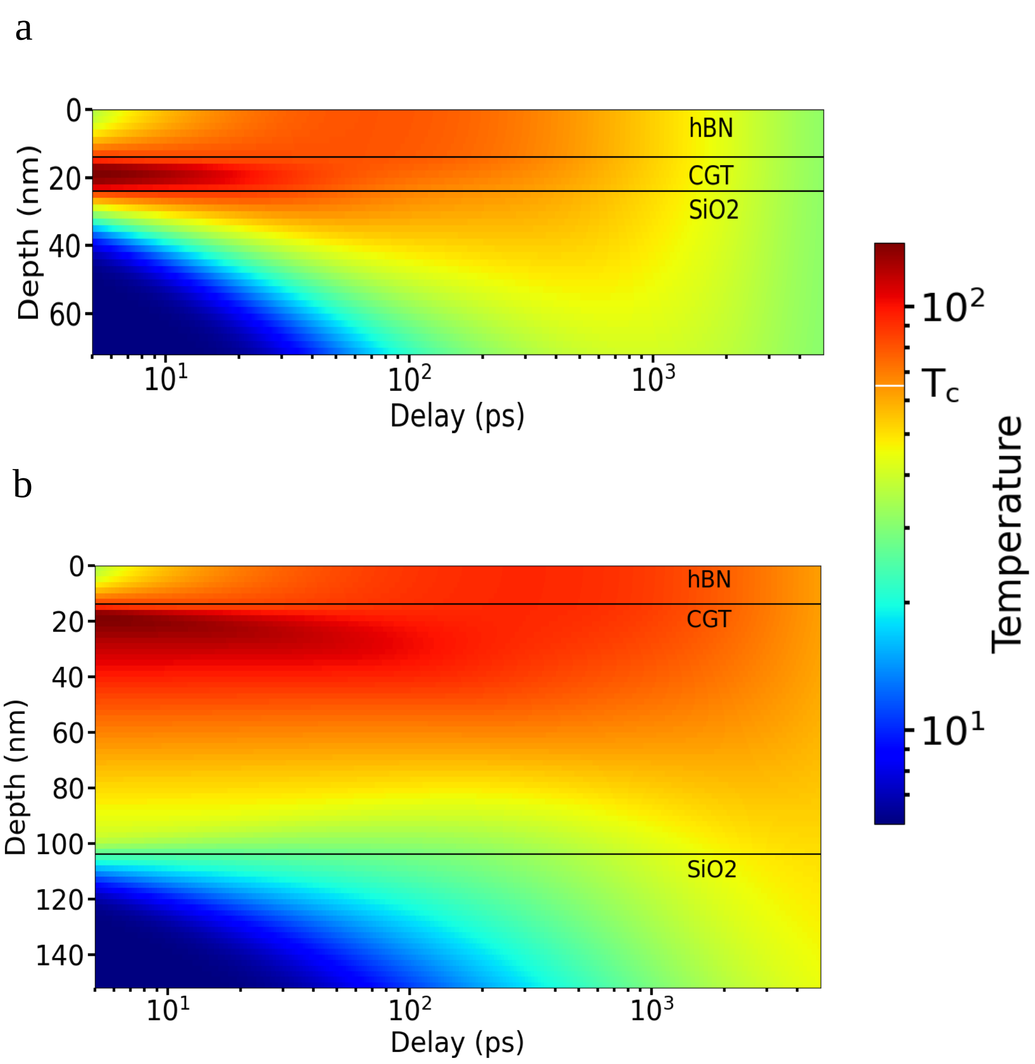

Figure 13: Phonon temperature dynamics induced by a laser pulse with fluence $0.5\mathrm{mJ/cm^2}$ for 10nm (a) and 90nm (b) thick CGT at temperature 6K. The thickness of hBN and SiO_2 are 14nm and 300nm, respectively. The Curie temperature T_c is highlighted in the color bar.

7 Additional data for magnetization dynamics from simulations

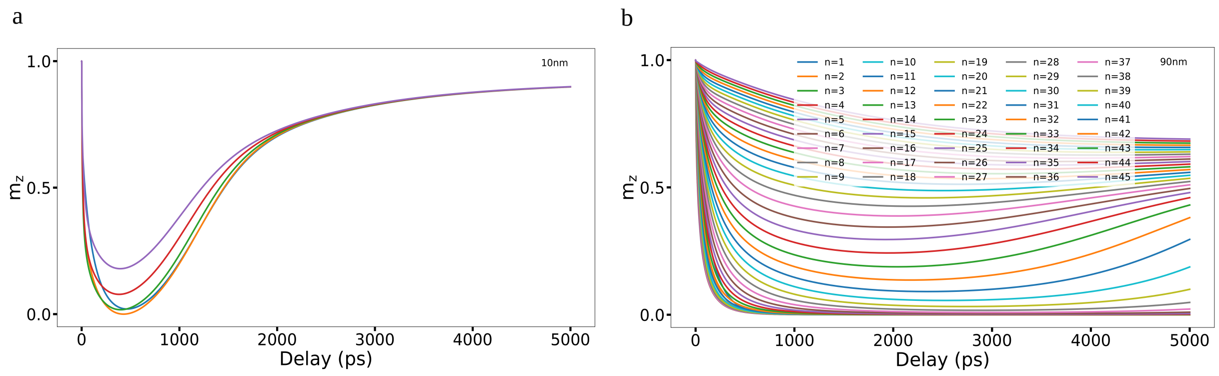

Figure 14: **a,** Normalized magnetization for 10nm (a) and 90nm (b) thick CGT samples after excitation of laser pulse with fluence $0.5\mathrm{mJ/cm^2}$. The thickness of hBN and SiO_2 are 14nm and 300nm, respectively.

8 Effect of the hBN thickness on the simulated magnetization dynamics

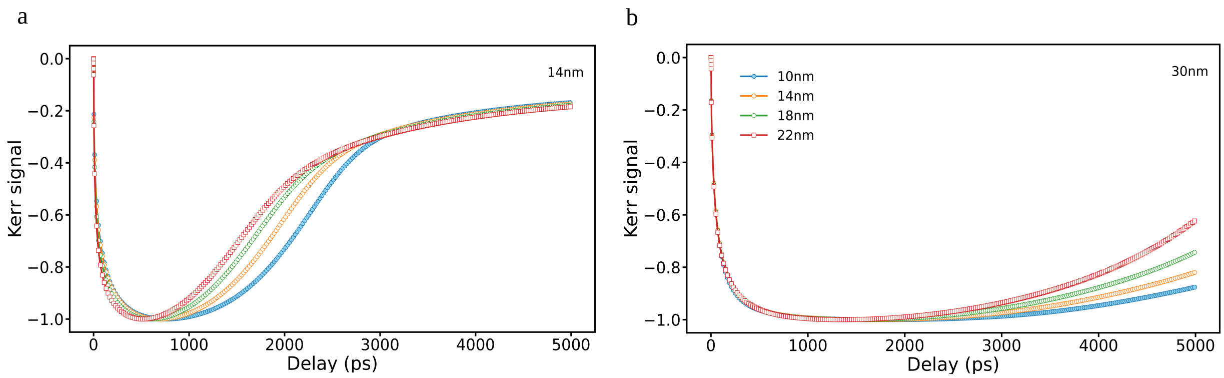

Figure 15: Evolution of Kerr signal for different thicknesses of hBN (10nm, 14nm, 18nm, and 22nm) for 14nm (a) and 30nm (b) thick CGT. The thickness of SiO_2 is 300nm for all the calculations.

9 Additional time-resolved measurements on the CGT crystal

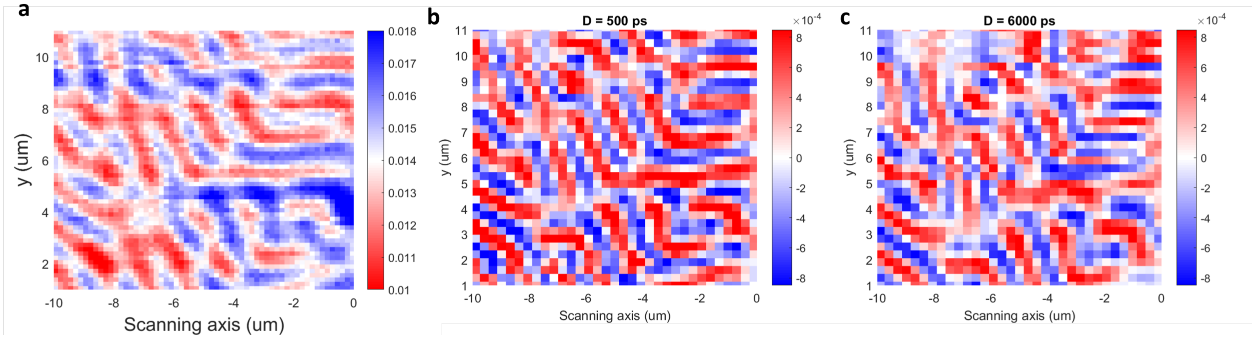

Figure 16: **a,** Static and **b,c** time-resolved MOKE images at remanence acquired at time delays 500 ps (**b**) and 6000 ps (**b**) with × 20 objective lens from the same area of the CGT crystal as in (**a**). H = 0 T, T = 6 K. Small shift of the pattern between (**b**) and (**c**) is due to the drift of the sample with respect to the probe beam.

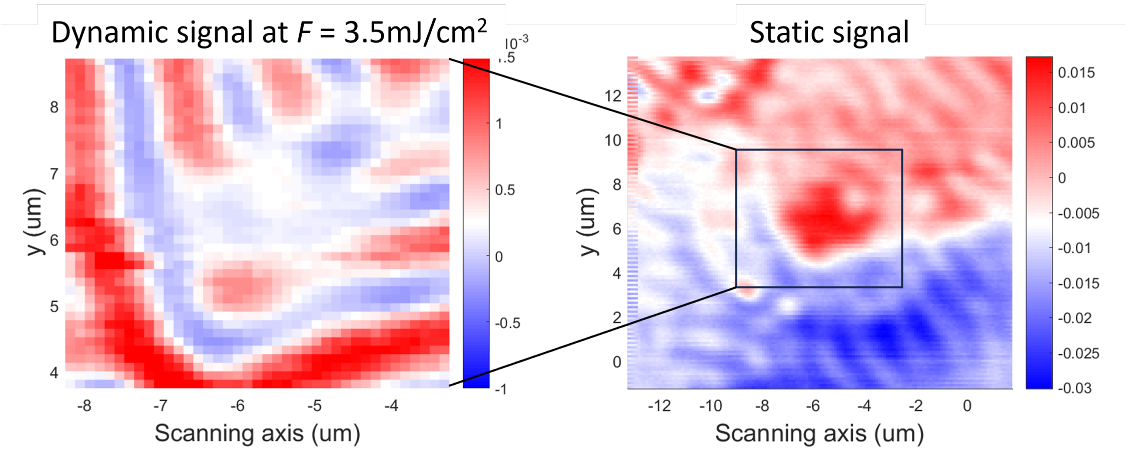

Figure 17: Effect of pumping with the intense pump beam during time-resolved measurements (**a**), as recorded by static MOKE image (**b**) following time-resolved measurements and after cycling the magnetic field to saturation (H = 0.5 T) and back to remanence (H = 0 T). Circular shape in the centre of the image (**b**) indicates permanent change to the magnetic ground state of the sample due to the pump beam. Images were acquired with × 20 objective lens from the same area of the CGT crystal. H = 0 T, T = 6 K.

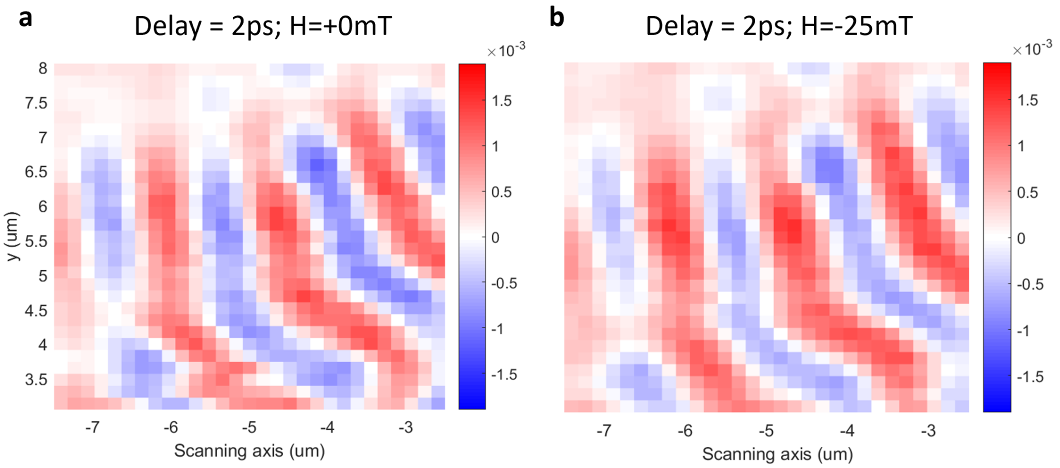

Figure 18: Time-resolved MOKE images at remanence (**a**) and -25 mT bias field (**b**) at 2 ps time delay acquired with $\times$ 20 objective lens. $T = 6$ K.

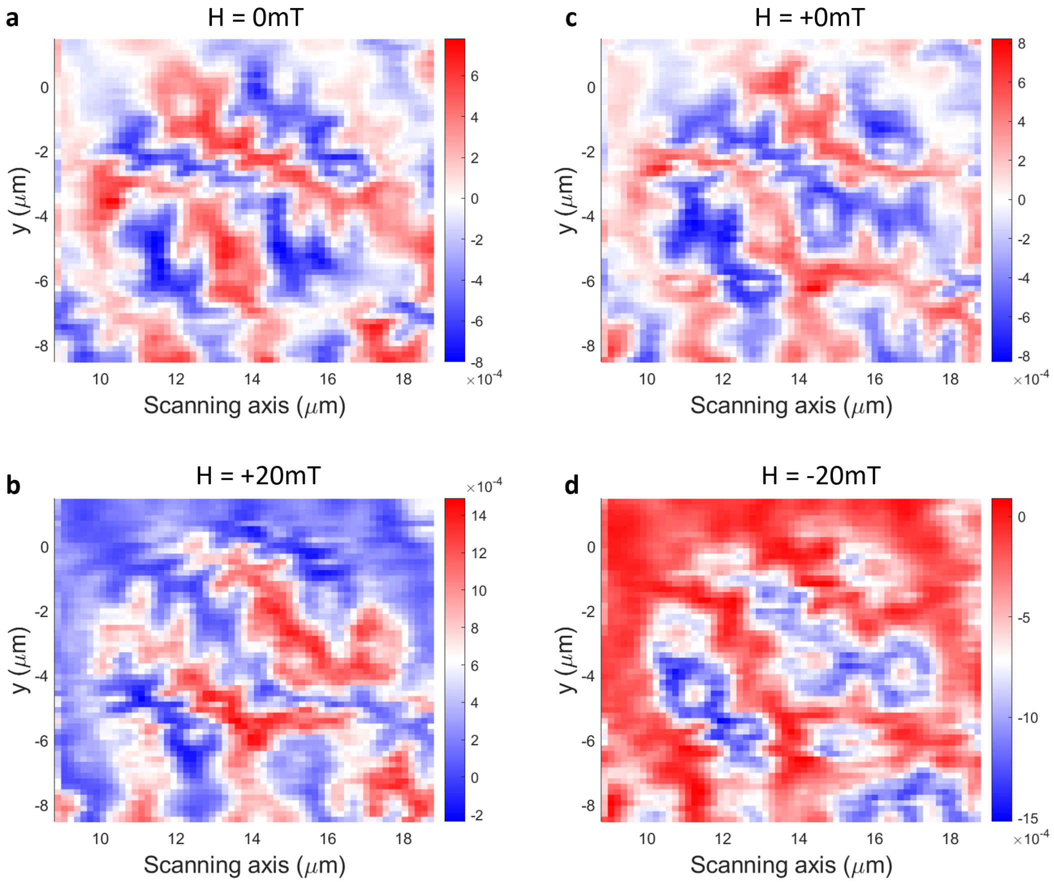

Figure 19: Time-resolved MOKE images at remanence and different bias fields and at 1000 ps time delay acquired with $\times$ 20 objective lens. $T = 6\,K$.

10 Non-thermal effects on ultrafast spin dynamics in CGT

In this section we will discuss additional effects arising from the non-thermal nature of spin excitations during the ultrafast demagnetization and remagnetization processes. We note that non-thermal nature stands for the fact that the distribution of magnetization does not follow a thermal distribution $f(\mathbf{S}_i) \sim \exp(-\mathscr{H}(\mathbf{S}_i)/k_\mathrm{B}T_e)$. It shall be stated here that the only difference in the dynamics, both of angular momentum and energy, of samples with different $CrGeTe_3$ (CGT) thicknesses we have found and modelled are driven by thermal diffusion effects. However, the temperature dynamics and profiles arising from different thermal diffusion properties of these samples have a highly non-linear effect on the non-thermal spin dynamics in CGT. Here, we demonstrate that the magnetization dynamics stem from a non-thermal spin distribution on nanoscale timescales, which have important implications on a deeper understanding of the optical excitations.

Figure 20 visualizes the non-thermal nature of the spin-excitations. Here, the top panels compare the magnetization dynamics of simulations conducted with 15 nm CGT (left) and 90 nm CGT (right). The grey curves refer to the respective mean field magnetization values computed from the respective electron temperature dynamics. The mean field equilibrium magnetization at a given temperature can be found by solving the self-consistent equation:

$$m_\mathrm{eq} \stackrel{!}{=} B_S(T, m_\mathrm{eq}), \tag{1}$$

where $B_S = \frac{2S+1}{2S}\coth(3\frac{2S+1}{2S}\frac{T_C}{T_e}m) - \frac{1}{2S}\coth(\frac{3}{2S}\frac{T_C}{T_e}m)$ is the Brillouin function for a given value of effective spin, evaluated at electron temperature T_e and magnetization m. The equilibrium magnetization plummets instantly once the electron temperature is excited above the Curie temperature

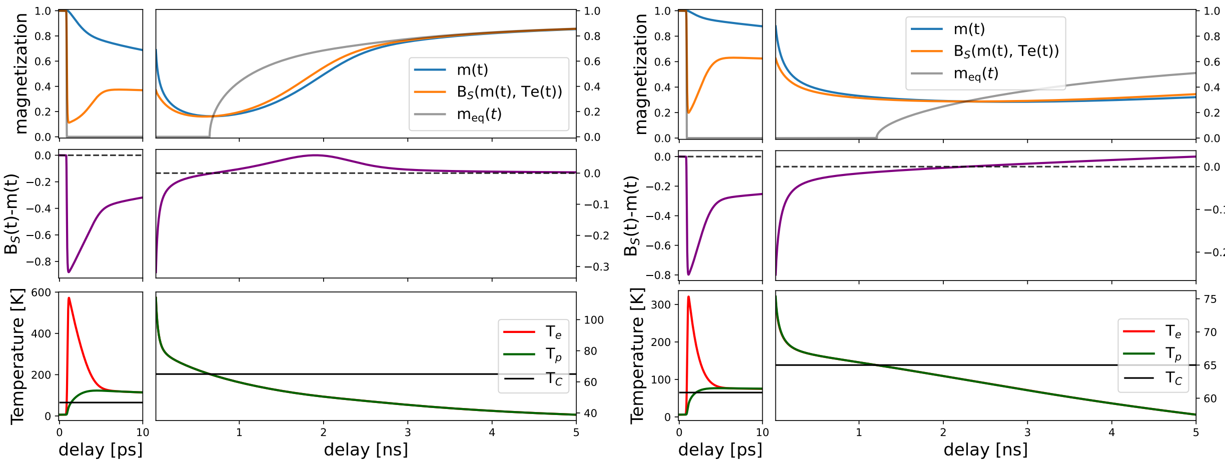

(a) 15 nm CGT

(b) 90 nm CGT

Figure 20: Visualization of the non-thermal spin dynamics in CGT. The left panel shows simulation data of a hBN/CGT/SiO_2 sample with 15 nm CGT, the right one shows an equivalent setup with 90 nm CGT. The top panels show the spatially averaged normalized magnetization dynamics $m(t)$ of CGT computed from the layer-resolved microscopic three temperature model (M3TM), the Brillouin function B_S and the equilibrium magnetization $m_{\text{eq}}(t)$. The middle panels show the difference of $m(t)$ and $B_S(m(t), T_e(t))$, which drives the magnetization dynamics. The bottom panels show the evolution of electron- and phonon-temperatures (T_e, T_p) over time, the Curie temperature (Tc) is marked for reference.

T_C (bottom panel) and starts recovering once the temperature drops below T_C. The magnetization starts to recover once $m_{\text{eq}}(t) > m(t)$. The difference $m_{\text{eq}}(t) - m(t)$ is a measure of how far the spin system is from thermal equilibrium with the electrons. The magnetization dynamics however are driven by the difference:

$$\delta m = m(t) - B_S(m(t), Te(t)), \tag{2}$$

as can be readily seen from the M3TM rate equation for spin $S = 1/2$:

$$\frac{dm}{dt} = Rm\frac{T_p}{T_C}(1 - m\coth(\frac{mT_C}{T_e})), \tag{3}$$

where $\tanh(\frac{mT_C}{T_e}) = B_{1/2}(\frac{mT_C}{T_e})$. Expanding m linearly in $B_S(m, Te)$ one finds:

$$\frac{dm}{dt} \propto -\delta m \tag{4}$$

Assuming thermalized electron- and spin-systems, one would expect the curves $m(t)$ and m_{eq} to be identical. Because the M3TM (just as Landau-Lifshitz formalism) assumes a finite rate of angular momentum dissipation that governs the magnetization dynamics, the magnetization lags behind the equilibrium value, evidencing the non-thermal nature of spin excitations. The M3TM assumes this is governed by a finite rate of spin flips during electron-phonon scattering events, a process induced by a prolonged lifetime of photo-excited electrons in the conduction bands of CGT[8]. The difference $-\delta m$ (Eq.(2)) is shown in the middle panel. It peaks at its minimum right after laser excitation and recovers as the spin system slowly equilibrates with the electronic system heated above T_C. As the temperature crosses T_C, the equilibrium magnetization increases rapidly. Again, the magnetization reacts slowly to the rapid change in the instantaneous equilibrium value and the dif-

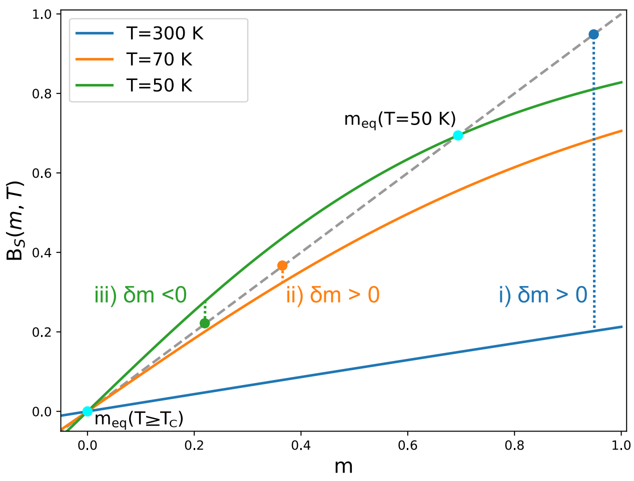

Figure 21: Brillouin functions B_S over magnetization for three values $T_e(t)$ (solid colored lines), showing the distinction of the three phases of magnetization dynamics. The dashed grey line corresponds to $m = m$, the colored dots denote three exemplary instantaneous magnetization values. i) A hot electron system drives the system far from equilibrium. ii) After electron-phonon equilibration the temperature close to T_C leads to slow demagnetization. iii) As soon as $m_{\text{eq}}(t)$ (light blue dots) $> m(t)$, the system remagnetizes.

ference $m_{\text{eq}} - m(t)$ changes sign. At long times, the magnetization will coincide with its equilibrium value, as can be seen in the 15 nm CGT sample in Figure 20. It is noteworthy that thermalized spins would be given by $m = B_{1/2}(\frac{mT_C}{T_e})$. One has to remember what is the meaning of the Brillouin function for $S = 1/2$, $Z = \sum_{m=-1/2,1/2} \exp(-E_m/k_B T)$, and the magnetization $m \sim \partial Z/\partial(1/T)$, here E_m is the MFA energy for the central spin, $J_0 m$. Thus, no-thermalize means that one can not cast Z and therefore m as a thermal distribution of over the energy but it follows some other non-equilibrium-thermal distribution. A visual representation of the magnetization rate is shown in Figure 21. Here, we highlight three distinct phases in the magnetization dynamics: *i)* right after pulse absorption, the electron system is heated up far beyond T_C, while the spin system is still very much ordered, leading to a large discrepancy $\delta m > 0$ and rapid demagnetization. *ii)* After electron-phonon equilibration the temperature bath is in close proximity to T_C while the magnetization is already significantly quenched, leading to a small rate $\delta m > 0$, marking the observed Type II dynamics. *iii)* On ns timescales, the temperature has dropped below T_C and again, the instantaneous magnetization reacts slow to the sudden change in m_{eq}, leading to remagnetization as the sign switches ($\delta m < 0$). As can be seen in the bottom panels of Figure 20, the average temperature in the thin CGT sample decreases more rapidly than in the thick CGT sample, which leads to a stronger remagnetization. However, also thermal equilibrium between electron- and spin-systems is reached quicker in this sample, although the magnetization rate parameters in both samples are exactly the same. This is due to the temperature dependence of the magnetization rate. As can be seen in Figure 22, the magnetization rate dm/dt is very low for temperatures close to T_C (black solid line) and magnetization values

of up to 0.4 due to the proximity of the instantaneous magnetization to thermal equilibrium. To visualize the difference in the dynamics we show the $m(T(t))$ curves from simulations the graphic, starting from the time when the temperature drops below T_C. While the thin sample (purple solid line) is quickly cooled to temperatures well below T_C and thus can quickly recover to its mean field equilibrium value, the temperature of the thick sample (magenta solid line) stays in close proximity to the Curie temperature during the whole 5 ns. It thus experiences a very small remagnetization rate and does not equilibrate with the electronic system on this timescale.

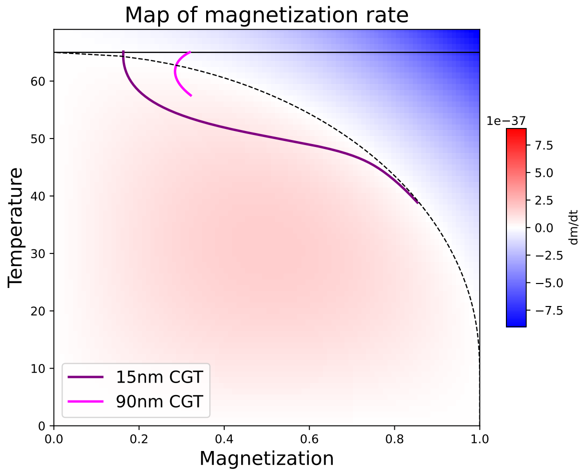

Figure 22: Magnetization rate dm/dt of CGT approximated with the M3TM for Spin $S = 1/2$ for temperatures close to and lower than $T_C = 65$ K, marked by the solid black line. The colored lines show the simulation data of thin and thick CGT samples after T_C-crossing. The dashed line represents the equilibrium magnetization.

For completeness, we shall mention that the color map of magnetization rate shown in Figure 22 was calculated for spin S=1/2 from Eq.3, where we used that electron- and phonon-temperatures are in equilibrium at the timescales of interest here. The parameter R was chosen the same as in the simulations shown in Figure 20. We noticed that the limited cooling in the thick sample has not only influence on the magnetization recovery rate, but also on the spin thermalization time. Importantly, this effect has significant physical consequences that go beyond the control of remagnetization timescales by varying the thickness

of CGT. The difference of instantaneous magnetization and equilibrium magnetization gives rise to a transient spin accumulation[9] given by:

$$\Delta\mu_s \propto dm/dt, \tag{5}$$

which is only driven as long as the spin system is not thermalized, as can be seen in Eq.(2) and Eq.(4). This spin accumulation can for instance generate a spin-polarized current, if the magnetic

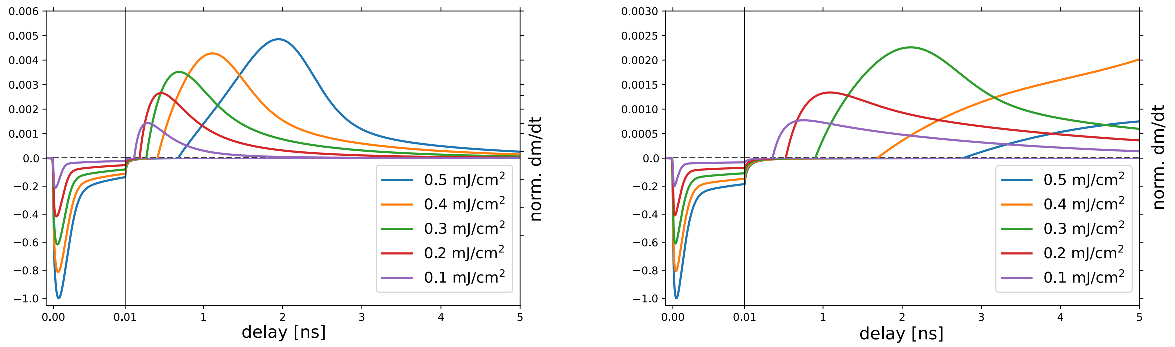

(a) 15 nm CGT

(b) 90 nm CGT

Figure 23: dm/dt signal for varying fluences, normalized to maximum demagnetization rate of the highest fluence value, representing the generated spin accumulation from the magnetization dynamics. The y-axis have been re-scaled to differentiate the ns dynamics during remagnetization.

material is interfaced by a normal magnet. In magnets that show Type I magnetization dynamics upon ultrafast laser irradiation this process is used to stimulate and control THz electric field emission in F/N heterostructures [9,10]. Our data and simulations suggest that the shape and amplitude of the spin accumulation and thus spin-polarized currents can be controlled by merely the thickness of the magnetic constituent in 2D van der Waals heterostructures and stimulate GHz electric field emission.

To further underline the controlability of the generated spin accumulation, we show fluence dependence of the magnetization rates in Fig. 23 for 15 nm and 90 nm CGT thicknesses. While for lower fluences, the expected spin accumulations are very similar in shape, the dynamics strongly diverge at the fluence of $F = 0.4$ mJ/cm^2, which marks the transition where electrons and phonons equilibrate at temperatures above T_C. Because of the different diffusion properties and the reaction of the magnetic subsystems described above, the thermalization times and thus spin accumulation and spin polarized currents diverge for higher fluences. This demonstrates a simple and straight-forward control of spin current generation by thickness control of CGT.

11 Ultrafast control of spin dynamics via thermal conductivity of a substrate

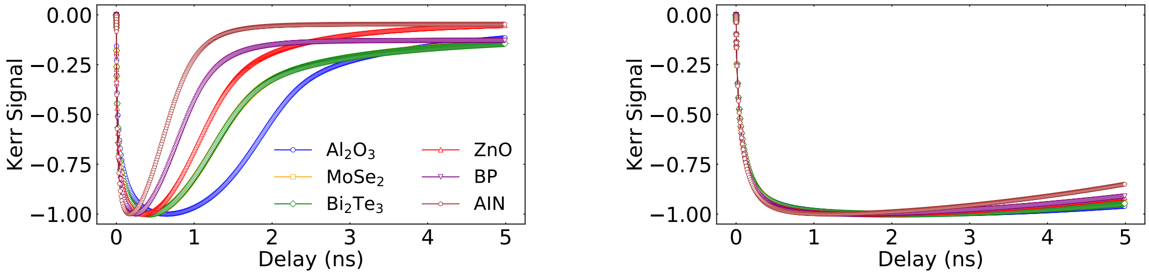

(a) 14 nm CGT interfaced with different substrates as indicated.

(b) Similar as (a) but 90 nm CGT.

Figure 24: Kerr signal calculated for six different substrates using similar approaches as those included in Figure 2 in the main text for two different CGT thickness, 14 nm (a), and 90 nm (b).

1. G. Ghosh Dispersion-equation coefficients for the refractive index and birefringence of calcite and quartz crystals. *Opt. Commun.* **163**, 95-102, (1999).

2. Idzuchi, H., *et al.* On the Optical Properties of $Cr_2Ge_2Te_6$ and Its Heterostructure. *Condens. Matter* **8**, 59, (2023).

3. Lee, S.-Y., *et al.* Refractive Index Dispersion of Hexagonal Boron Nitride in the Visible and Near-Infrared. *Phys. Status Solidi B* **256**, 1800417, (2019).

4. Hamrle, J., Pištora, J., Hillebrands, B., Lenk, B. & Münzenberg, M. Analytical expression of the magneto-optical Kerr effect and Brillouin light scattering intensity arising from dynamic magnetization. *J. Phys. D: Appl. Phys.* **43**, 325004, (2010).

5. Hamrle, J., Ferré, J., Nývlt, M. & Višňovský, Š In-depth resolution of the magneto-optical kerr effect in ferromagnetic multilayers. *Phys. Rev. B* **66**, 224423 (2002).

6. Atxitia, U., Chubykalo-Fesenko, O., Walowski, J., Mann, A. & Münzenberg, M. Evidence for thermal mechanisms in laser-induced femtosecond spin dynamics. *Phys. Rev. B* **81**, 174401 (2010).

7. Koji O. & Hatsuo I. Matrix formalism for calculation of electric field intensity of light in stratified multilayered films. *Appl. Opt.* **29**, 1952–1959, (1990).

8. Sutcliffe, E. *et al.* Transient magneto-optical spectrum of photoexcited electrons in the van der Waals ferromagnet $Cr_2Ge_2Te_6$. *Phys. Rev. B* **107**, 174432 (2023).

9. Reza, R. *et al.* Laser-induced terahertz spin transport in magnetic nanostructures arises from the same force as ultrafast demagnetization. *Phys. Rev. B* **106**, 144427 (2022).

10. Zhang, W. *et al.* Ultrafast terahertz magnetometry. *Nat. Commun.* **11**, 4247 (2020).
